# On-Surface Synthesis of a Low Bandgap Cyclopenta[*c*,*d*]azulene-Based Polymer

**DOI:** 10.1021/jacs.6c08825

**Published:** 2026-10-14

**Authors:** S. Jiménez-Fernández, F. Villalobos, V. Kumar, J. I. Martínez, J. I. Mendieta-Moreno, P. L. de Andrés, E. Tosi, J. A. Martín-Gago, J. M. Cuerva, I. Palacio, A. G. Campaña, C. Sánchez-Sánchez

**Affiliations:** † ESISNA Group, 69570 Instituto de Ciencia de Materiales de Madrid (ICMM-CSIC), Sor Juana Inés de la Cruz 3, Madrid 28049, Spain; ‡ Institute of Physics of the Czech Academy of Sciences, Cukrovarnická 112/10, Prague 6 162 00, Czech Republic; § Departamento de Química Orgánica, 16741 Universidad de Granada (UGR), Unidad de Excelencia de Química UEQ, C. U. Fuentenueva, Granada 18071, Spain; ∥ Departamento de Física Teórica de la Materia Condensada, 16722 Universidad Autónoma de Madrid, Madrid 28049, Spain

## Abstract

Nonbenzenoid topologies offer a compelling strategy to tailor the electronic properties of low-dimensional carbon materials. Here, we report the on-surface synthesis of a nonalternant polymer incorporating a [7–5–5] cyclopenta­[*c*,*d*]­azulene (CPA) motif on Au(111) via thermally induced Ullmann coupling. Bond-resolved scanning tunneling microscopy confirms the robustness of the [7–5–5] framework. Scanning tunneling spectroscopy, together with d*I*/d*V* mapping supported by density functional theory calculations, reveals a remarkable narrow polymeric bandgap of 1.5 eV. The electronic structure is governed by the intramolecular charge transfer from the heptagonal ring to the adjacent fused pentagonal rings, resulting in a pronounced dipole moment despite being composed exclusively of carbon and hydrogen. Aromaticity analyses indicate the coexistence of locally aromatic and slightly antiaromatic regions, highlighting the intrinsic electronic frustration associated with the nonalternant topology. These findings demonstrate how the controlled incorporation of [7–5–5] motifs enables the rational bandgap engineering in carbon-based polymers and expands the scope of nonbenzenoid design in on-surface synthesis.

## Graphical abstract

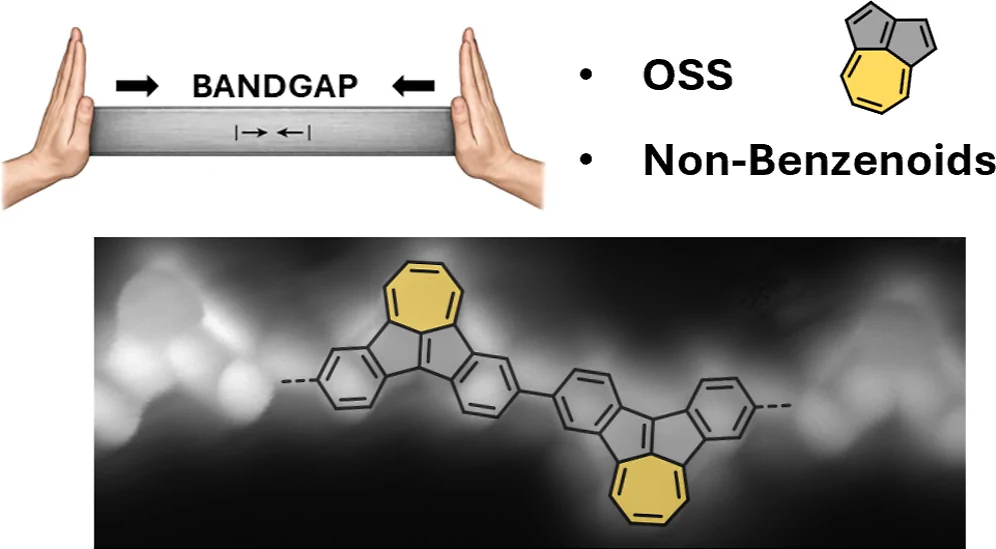

## Introduction

Over the past years, the deliberate incorporation of nonbenzenoid rings into graphene-like low-dimensional materials has emerged as a strategy for tailoring their properties.^1^ The resulting disruption of the intrinsic lattice symmetry enables new structural and electronic functionalities, modifying aromaticity, and packing, enhancing charge mobility or improving optical and/or magnetic properties.^2,3^ The careful selection of the number, position, and type of nonbenzenoid rings drastically influences the material’s properties by modulating both strain (planar versus curved architecture) and electronic structure. Some clear examples are Haeckelite tubes, a nonalternant carbon nanotube that has been theoretically predicted to show superconductivity, high-temperature supercurrents, or metallic properties with closed bandgaps;^4^ Schwarzites, considered as “the holy grail” of carbon allotropes for its unique predicted properties;^5^ and the biphenylene network, a nonbenzenoid graphene allotrope,^6^ as well as biphenylene graphene nanoribbons^7^ and nanosheets.^8^

One of the most iconic nonbenzenoid units incorporated in graphene-based frameworks is azulene (**1**; see Figure 1), the classical nonbenzenoid isomer of naphthalene. This pure hydrocarbon displays charge transfer from an electron-deficient seven-membered ring to a fused electron-rich five-membered ring, giving rise to a reduced HOMO–LUMO gap, a permanent dipole moment of 1.08 D,^9,10^ and a deep-blue color in comparison to colorless naphthalene (with a dipole moment of 0 D, no charge transfer, and an optical HOMO–LUMO gap over 4 eV).^11^ These unusual electronic characteristics have made azulene a widely used structural motif for tuning the electronic and magnetic properties, finding this motif within nanographenes, graphene polymers, or nanoribbons.^10,12–14^ A natural evolution of azulene is its expansion by the incorporation of an additional five-membered ring to its core, giving rise to a family of [7–5–5] systems that exhibit different electronic properties upon the relative orientation and position of these nonbenzenoid rings.^15^

**Figure 1:**
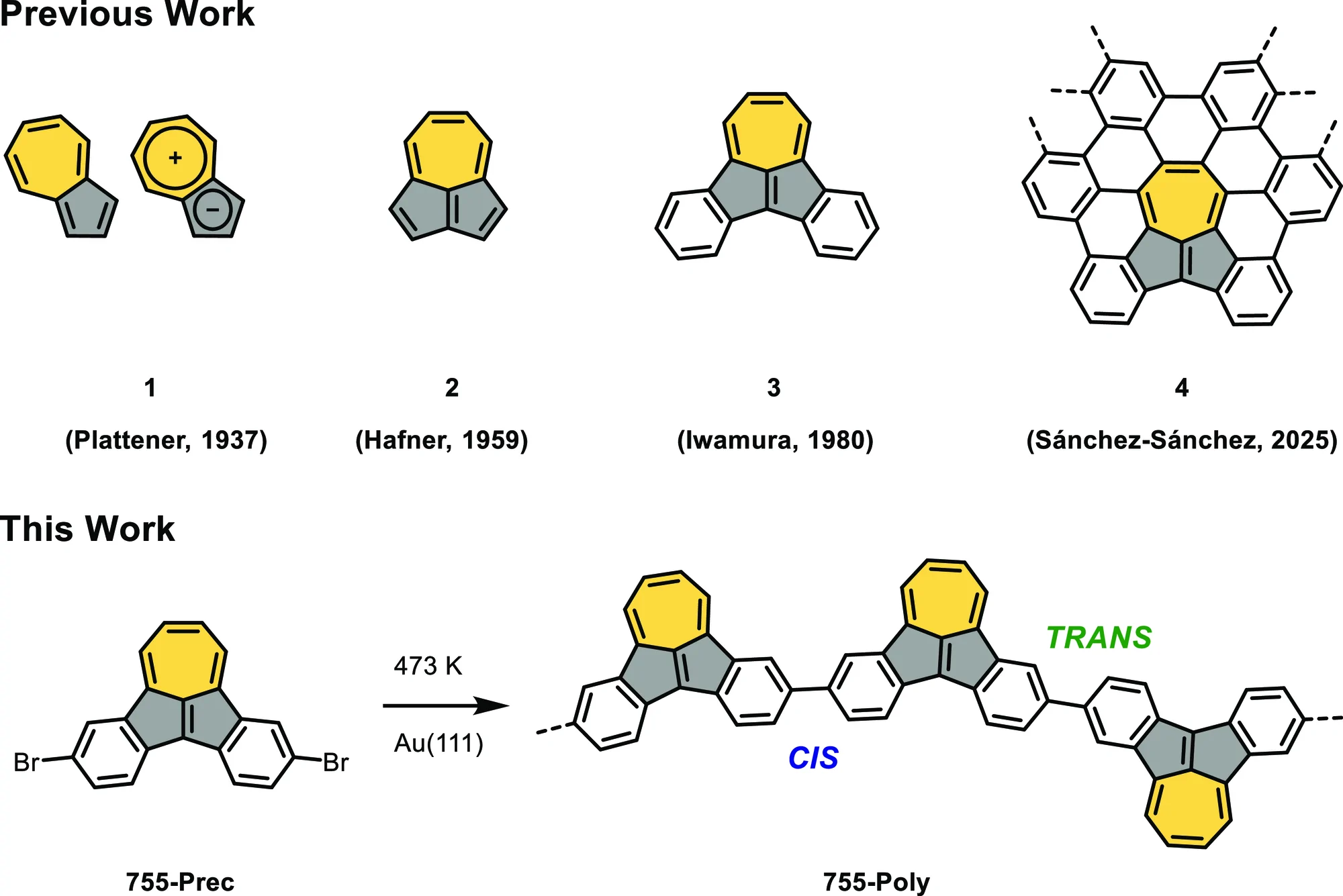
Top: scheme of relevant structures to this work. Bottom: on-surface synthesis of **755-Poly**.

Among these, cyclopenta­[*c*,*d*]­azulene (CPA)—a [7–5–5]-fused system consisting of two five-membered rings and a seven-membered ring sharing a central carbon atom (**2**, see Figure 1)—is of particular interest as it has already been shown its unique potential to lower the bandgap when incorporated into a chevron-like graphene nanoribbon.^16^ **2** was first synthesized by Hafner and co-workers in 1959,^17^ and its structure was conclusively established in 1970.^18^ Subsequently, the synthesis of the extended derivative (**3**) by Iwamura et al.^19^ was reported, as well as other metalated analogues.^20^ A related on-surface study demonstrated the generation and controlled structural isomerization of [7–5–5] motifs in one-dimensional molecular architectures.^21^ More recently, the on-surface synthesis of GNRs incorporating [7–5–5] units (**4**) by ring contraction of a benzannulated cyclooctatetraene (COT) polymer has been reported. These exhibit a narrow bandgap of 1.43 eV attributed to the distinctive electronic properties of the [7–5–5] motif.^16^ Related open-shell ring contraction of benzofused COT has also led to the formation of a sesquifulvalene moiety incorporating a similar [7–5–5] unit.^22^ In addition, [5–7–5] GNRs and [7–5–5] trimers have also been recently reported.^23,24^

Beyond these systems, the [7–5–5] motif can be found embedded in more complex molecular architectures, such as fullerenes,^25,26^ other graphene-like materials, or pharmaceutically relevant molecules and natural products,^27,28^ underscoring its broad structural and functional relevance. Together, these findings highlight the potential of this motif as a versatile platform for tuning the electronic properties. Nevertheless, reports on synthetic routes, reactivity studies, and electronic characterization employing this [7–5–5] motif to construct graphene-like nanostructures remain underexplored in the literature, with the systems reported so far relying on inefficient and/or unpredictable strategies.

In this work, we report the efficient, reproducible, and direct high-yield on-surface synthesis, under ultrahigh-vacuum (UHV) conditions, of a novel nonbenzenoid polymer (**755-Poly**, Figure 1) incorporating [7–5–5] cyclopenta­[*c*,*d*]­azulene moieties. The polymer, so far not reported in solution, is obtained via thermal polymerization of the precursor **755-Prec** (Figure 1) (6,11-dibromobenzo­[*a*]­indeno­[1,2,3-*cd*]­azulene) on a Au(111) surface. The molecular structure is unambiguously characterized by bond-resolved scanning tunneling microscopy (BR-STM) using CO-functionalized tips, demonstrating the thermal stability of the [7–5–5] motif on Au(111). A comprehensive investigation of the electronic properties using scanning tunneling spectroscopy (STS) and d*I*/d*V* mapping, supported by density functional theory (DFT) calculations, reveals a significant reduction (∼20%) of the bandgap when compared to, for example, azulene-based polymers (1.5 eV vs 1.8 eV).^13^ Furthermore, aromaticity analysis based on NICS and anisotropy of the induced current density (ACID), together with distribution charge calculations, indicate the coexistence of locally aromatic and weakly antiaromatic regions within the polymer, arising from charge redistribution within the [7–5–5] motif. Overall, this synthetic strategy provides a promising route to low-dimensional materials with intriguing tunable optoelectronic properties through the incorporation of nonbenzenoid moieties.

## Results and Discussion

The deposition of **755-Prec** onto the Au(111) surface at room temperature results in the formation of a porous supramolecular network (Figure S1, Supporting Information), where each pore is formed by six monomers. This self-assembled phase is stabilized by triple nodes of Br-substitution, forming a cyclic structure. This triple node of Br atoms has been reported in previous studies.^29^ Importantly, there is no preferential distribution of the molecules forming the pores with consecutive molecules presenting either the same or opposite orientations.

After characterizing the self-assembly, **755-Poly** was synthesized on the surface either by subsequent postannealing at 473 K or by direct sublimation of **755-Prec** onto a clean Au(111) surface held at this temperature. Figure 2a shows the formation of long (Figure S2), high-quality linear structures mainly following the fcc regions of the herringbone reconstruction of the Au(111) surface thanks to the adaptability offered by the two possible coupling configurations (*cis* introducing curvature and *trans* being linear, as will be discussed below). The occurrence of the Ullmann-type coupling was confirmed by the analysis of the periodicity along the polymers, which yields a value of 1.0 ± 0.1 nm, consistent with the theoretical value of 0.94 nm for the periodicity along covalently linked molecular units. Figure 2b shows that coupling between **755-Prec** units can proceed via two configurations: *cis* or *trans* (see blue and green arrows, respectively; see also Figure 1 bottom panel). The *cis* configuration is responsible for the appearance of curvature in the polymer, as the two activated C atoms in each molecule subtend an angle of around 168°, resulting in an accumulated deviation of approximately 12° from the linear coupling. In contrast, the *trans* coupling compensates for this angular mismatch, yielding linear sections within the polymers. It is worth mentioning that our statistical analysis on the abundance of *cis* vs *trans* coupling configurations performed over 1500 molecular units (Figure S3 in Supporting Information) indicates that they are almost equally probable on the surface but for a slight preference for the *cis* one. We attribute this to the templating effect played by the herringbone reconstruction as the *cis* configuration allows the polymer adapting to the herringbone kinks and stay on the fcc regions. Additionally, we have studied if there is any clear sequence in the molecular configuration along the polymers to see if the molecular dipole moment can play a role on it. For this purpose, we have performed a correlation factor analysis along the different polymers formed by the 1500 molecular units mentioned above. In this analysis, we assign a value of 0/1 to the two different molecular configurations along each polymer and get the correlation factor as indicated in the Section S4 of the Supporting Information. A final value of −1/+1 means that all molecular units along each polymer are *trans*/*cis*, while a value of 0 means that the polymer growth is stochastic. In our case, we obtain values of 7.0 × 10^–2^ and 4.0 × 10^–2^, i.e., ∼0, for first and second neighbors, respectively, clearly indicating that the orientation of the last unit in the polymer does not guide the orientation of the next molecule attaching to it. Thus, it is reasonable to assume that the large intrinsic dipolar moment of the molecule (1.69 D)—comparable to that of iodobenzene (1.7 D)^30^ and water (1.85 D)^31^—does not play a pivotal role in orienting the monomers during the on-surface polymerization. This behavior can be rationalized in terms of the interaction between the molecule dipoles and their induced image dipoles in the metal, which effectively suppress dipole–dipole interactions between molecules oriented parallel to the surface.^13^ At this point, it is interesting to theoretically analyze the dipole moment of other fused [5–7–5] configurations. As can be observed in Figure S4, the position of the fused pentagon–heptagon–pentagon has great influence on the net dipole moment. Thus, combinations with the two pentagonal rings no longer fused, but separated by one (**6**, Figure S4)^22^ or two C–C bonds (**5**, Figure S4)^23^ present a much lower net dipole moment as the charge transfer from the heptagon toward each flanking pentagon results in a partial cancellation of the polarization vectors.

**Figure 2:**
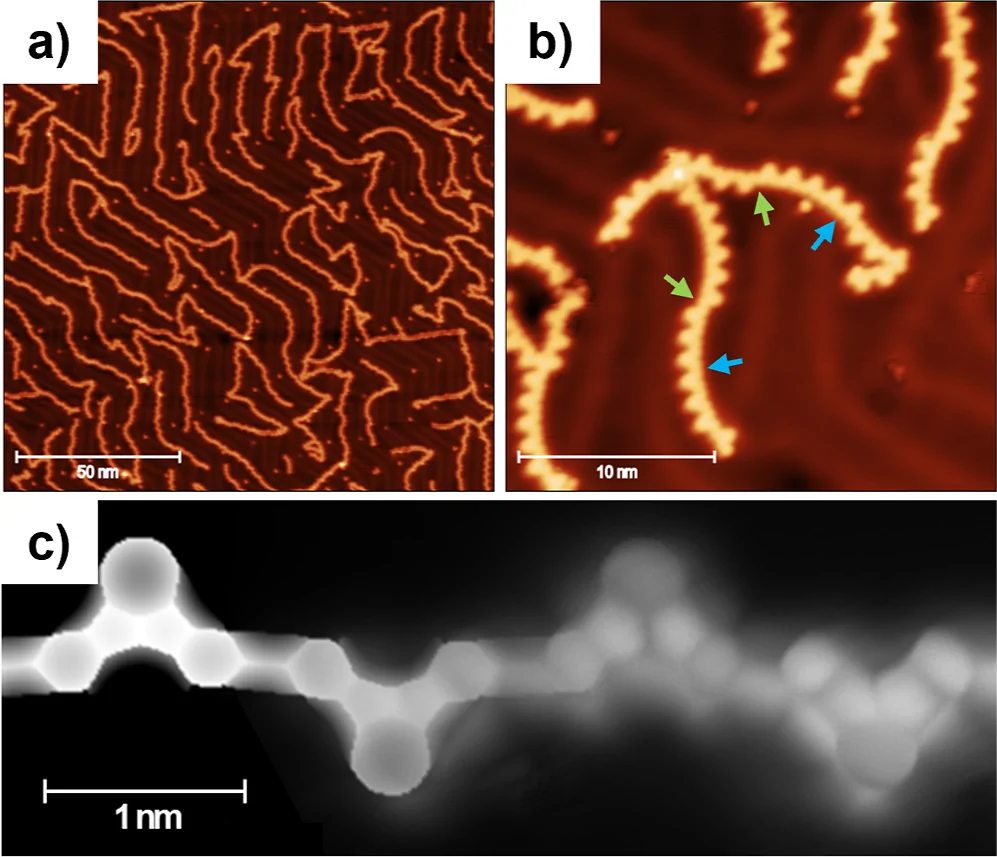
On-surface synthesis of **755-Poly**. (a,b) STM topographic images of **755-Poly** with a CO-functionalized tip after **755-Prec** evaporation with the substrate held at 473 K (*V*_bias_ = 130 mV, *I*_t_ = 100 pA). (c) BR-STM image with a CO-functionalized tip (*V*_bias_ = 2 mV). Δ*Z* = −0.18 nm; the feedback loop was opened at *V*_bias_ = 130 mV, *I*_t_ = 20 pA, with the tip positioned above the seven-membered ring. Simulated BR-STM image superimposed at the left part of (c). The CPA moiety is clearly identified with one heptagon and two pentagons.

Figure 2c presents a bond-resolved STM image acquired using a CO-functionalized tip, with the simulated BR-STM image superimposed. The [7–5–5] cyclopenta­[*c*,*d*]­azulene moiety is clearly resolved, demonstrating its thermal stability at temperatures even higher than those of the Ullmann reaction (573 K).

Subsequently, the electronic properties of **755-Poly** were investigated using STS spectroscopy, d*I*/d*V* mapping, and theoretical calculations. Figure 3a shows two spectra (see Figure S5 for an extended version of the spectra): one acquired on the Au(111) substrate and the other on **755-Poly** (positions are color-coded in the STM image inset). Two main resonances at −400 and 1100 meV were identified in **755-Poly**, revealing a bandgap of 1.5 eV, narrower than that observed, for example, for other azulene-based polymers (1.8 eV)^13^ and rather uncommon within polymeric nanostructures.

**Figure 3:**
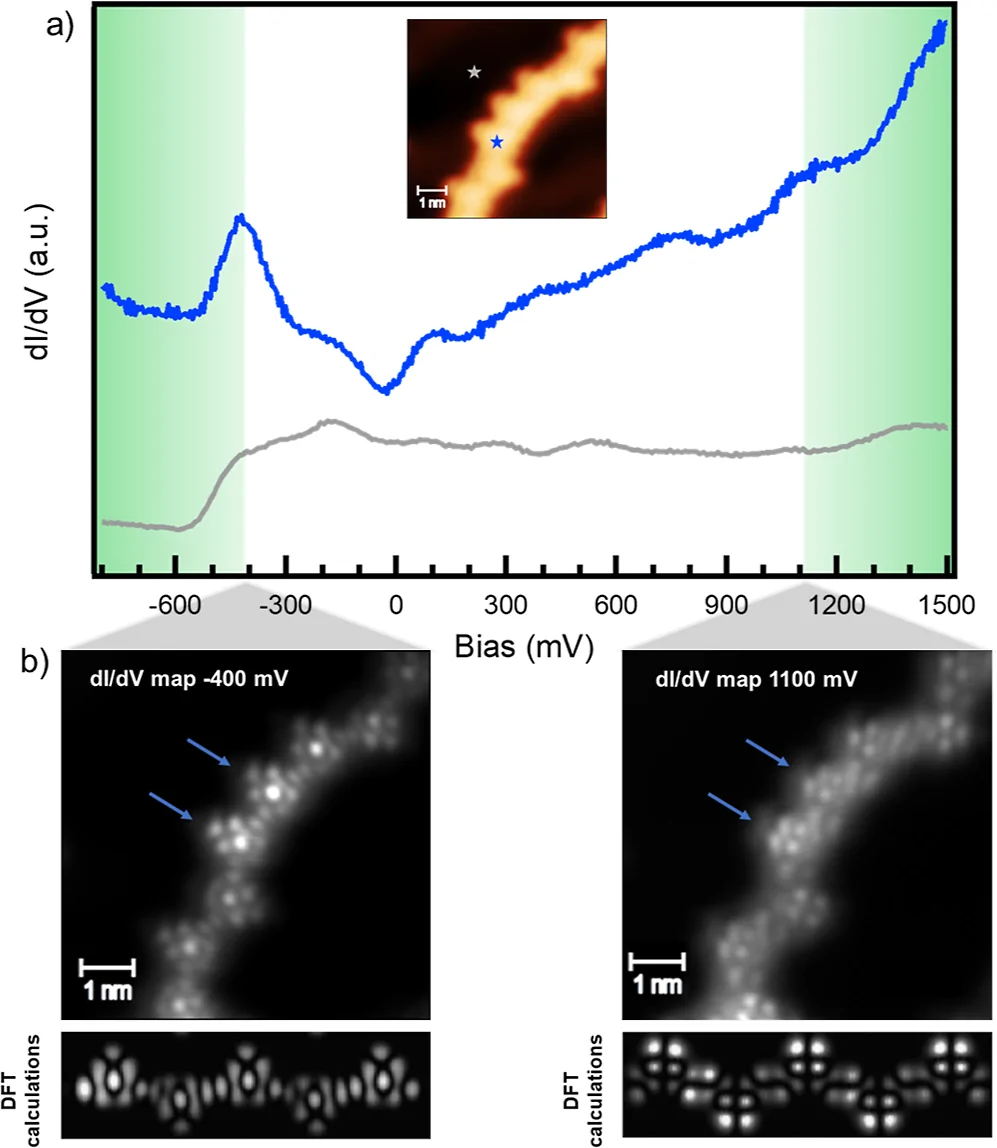
Electronic characterization of the **755-Poly**. (a) STS spectra of **755-Poly** on Au(111) (position of the spectra is color-coded in the STM image of the inset, *V*_bias_ = 300 mV, *I*_t_ = 100 pA). (b) d*I*/d*V* maps of the two main resonances at −400 mV and 1100 mV, including the d*I*/d*V* simulated maps of the HOMO and LUMO for the trans configuration. The molecular units marked by the blue arrows present a higher contrast due to the corrugation of the underlying herringbone-reconstruction of the Au(111) substrate. Lock-in parameters: *V*_ac_ = 50 mV, *f* = 769 Hz.

This assignment is corroborated by d*I*/d*V* maps acquired at these positions, where the two resonant states are clearly resolved (see Figure S6), in excellent agreement with the DFT-simulated HOMO and LUMO d*I*/d*V* maps (Figure 3b). Notably, although a feature is observed in the STS curve at around 750 mV, we attribute it to the onset of the LUMO as its corresponding d*I*/d*V* map does not clearly resolve the characteristic internal features. Indeed, these only become clearly apparent at 1100 mV (see Figure S6). It is interesting to note that the electronic properties are independent of whether the intermolecular coupling adopts a *cis* or *trans* configuration, as evidenced by the STS measurements carried out on molecular units with different configurations and by theoretical molecular orbitals and band diagrams (see Figure S7). Molecular units exhibiting higher contrast, highlighted by blue arrows in Figure 3b, arise from the corrugation of the herringbone reconstruction of the underlying Au(111) substrate (see Figure S8). At the −400 mV resonance, an enhanced contrast is observed at the center of each unit, reflecting the fact that the electronic density of this state is predominantly localized in the region between the two pentagons. In contrast, the state at +1100 mV displays a more uniform contrast along the polymer backbone, with the heptagon region splitting into two lobes, which are also visible in topographic STM images acquired at high positive bias voltages (see Figure S9). Notably, our bond length analysis based on our theoretical calculations indicates a value of 1.47 Å between adjacent molecular units, much longer than that obtained for a double (1.33–1.35 Å) and a triple bond (1.20–1.22 Å),^32,33^ but slightly shorter than that of prototypical single bonds in ethane (1.53 Å). Thus, we can unambiguously state that adjacent units along the polymer are connected through a single-like bond, limiting the conjugation along, resulting in a significantly reduced electronic coupling between neighboring units. Consequently, the spectroscopic features of the polymer closely resemble those of the gas phase molecule (see Figure S10), in agreement with similar observations reported in previous studies.^34^ Rather than diminishing the relevance of the extended architecture, this result indicates that the characteristic electronic features of the [7–5–5] unit are largely preserved upon incorporation into the surface-supported polymer, thereby allowing the polymer’s electronic properties to be rationalized from those of its individual building blocks. The narrow bandgap of **755-Poly** is tentatively attributed to the low HOMO–LUMO gap of the cyclopentaazulene unit that is incorporated into the polymer, which is given by the charge transfer process from the atoms in the seven-membered ring to those in the fused five-membered rings, also resulting in its intrinsic large dipole moment (1.69 D). Other effects like long-range polymer conjugation or substrate-induced electronic renormalization can be ruled, as increasing the length of the polymer only yields a modest reduction of the intrinsic molecular gap (see Figure S11 and discussion in Supporting Information), while our periodic DFT calculations show that the electronic structure is already essentially converged at the polymer limit and remains almost unchanged after adsorption on Au(111) (see Figure S12 and discussion in Supporting Information).

To determine the aromaticity of the molecule, we have performed nucleus-independent chemical shift calculations at 1 Å above the ring plane (NICS(1))^35^ and ACID^36^ analyses, together with a charge transfer calculation (Figure 4). The NICS (Figure 4a) shows negative values (blue regions) at the center of the hexagonal and heptagonal rings, indicating diatropic ring currents, a signature of aromatic systems. Conversely, the fused pentagons present slightly positive values (red regions), corresponding to paratropic currents, characteristic of antiaromatic systems with destabilizing electron delocalization. To contextualize this result, we have computed the NICS(1) for three related molecules: benzene, azulene, and pentalene (Figure S13). As we can see, our hexagonal rings present a smaller value than that of benzene (−1.3 vs 4.8 in benzene) and the value for our pentalene unit is also highly reduced with respect to the pentalene molecule (from +18.8 to +2.2). On the other hand, our heptagonal ring increases its aromaticity value with respect to that in azulene (−3.6 vs −2.9). To have the full picture about the aromatic behavior of the system, we have computed the ACID (Figure 4b), which indicates that the benzenoid rings and the heptagon present diatropic currents (blue arrows) while pentagonal currents are paratropic (red arrows). Altogether, it points toward an aromatic character of the hexagonal and heptagonal rings and a slightly antiaromatic character for the pentalene unit. Interestingly, in structurally related [5–7–5] systems where the pentagonal rings are not fused but separate by one or two C–C bonds, the central heptagons between pentagons are reported to present an antiaromatic character.^22,23^ This dichotomy underscores how the relative placement of nonbenzenoid rings tunes local aromatic/antiaromatic behavior.

**Figure 4:**
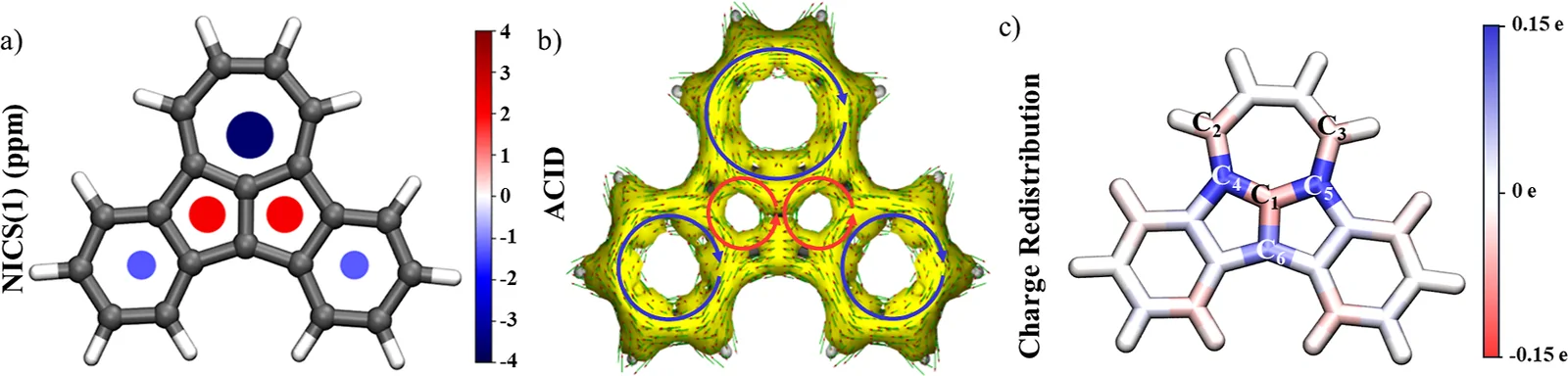
(a) Calculated nucleus-independent chemical shift (NICS(1)) of one unit of the **755-Poly** (gas phase). Blue regions represent aromatic behavior, while red regions, antiaromatic character. The heptagonal ring shows aromatic behavior, whereas the pentagonal rings exhibit mild antiaromaticity. (b) Calculated ACID pattern of one molecular unit, where blue arrows indicate aromatic diatropic and red arrows antiaromatic paratropic ring current, showing a good concordance with NICS results. (c) Calculated charge redistribution of one molecular unit. Blue regions indicate negative charge accumulation, while red regions indicate electron depletion.

Figure 4c illustrates the simulated charge redistribution within the polycyclic unit. The color scale ranges from blue (negative charge accumulation) to red (positive charging), with values being between approximately ±0.15 e. C_1_, C_2,_ and C_3_ atoms show a decrease of negative charge (red color), consistent with their role as electron donors to the neighboring C_4_, C_5,_ and C_6_ atoms, which act as electron acceptors (blue color). This charge redistribution reflects the electronic frustration inherent to nonalternant polycyclic systems, in which locally aromatic regions coexist with antiaromatic domains, as supported by NICS and ACID analyses. The competition between these opposing electronic characters often drives symmetry-breaking distortions, such as Jahn–Teller effects, which lift electronic degeneracy and provide stabilization.

## Conclusion

In conclusion, we report the on-surface synthesis of a novel nonbenzenoid polymer, incorporating a [7–5–5] cyclopenta­[*c*,*d*]­azulene moiety on Au(111). Statistical analysis shows no significant preference between *cis* and *trans* configurations along the polymers. STS measurements and d*I*/d*V* mapping, in agreement with DFT calculations, demonstrate a remarkable narrow bandgap of 1.5 eV for polymer, attributed to the incorporation of the cyclopentaazulene units with a small HOMO–LUMO gap, making this system appealing for applications in organic photovoltaics, photodetectors, and ambipolar field-effect transistors.^37–39^ Aromaticity (NICS and ACID) and charge redistribution calculations reveal the coexistence of aromatic and antiaromatic regions, typical of nonalternant hydrocarbon nanostructures. This work opens new avenues for the on-surface synthesis of organic nanostructures incorporating CPA motifs, thereby expanding the toolbox for nonbenzenoid molecular design.

## Experimental Section

STM images were acquired in a UHV chamber with a base pressure of 1.0 × 10^–10^ mbar, equipped with an LT-STM/ncAFM (Scienta Omicron GmbH) operated at 4.5 K and a LEED/AES (OCI Vacuum Microengineering Inc.). The Au(111) single crystal (Mateck) surface was prepared by repeated cycles of argon ion sputtering at 1.5 keV followed by annealing up to 723 K. The sample quality was checked by means of STM and/or LEED. Molecules were evaporated at 460 K (corresponding to an evaporation rate of 0.6 Å/min) from a quartz crucible installed in a 6-fold organic evaporator (Mantis GmbH). STM images were processed and analyzed with the Gwyddion software.^40^

Molecular calculations were carried out using Gaussian16 ^41^ (B3LYP/cc-pVTZ level).^42,43^ Periodic calculations were performed with Quantum Espresso^44^ by using the PBESol functional,^45^ RRKJ ultrasoft pseudopotentials,^46^ and DFT-D3 dispersion corrections.^47^ The unreconstructed Au(111) surface was modeled by considering a four-layer slab with the bottom two layers fixed, and a six-unit polymer fragment terminated with methyl groups was adsorbed on the surface with a vacuum region of at least 20 Å. A [1 × 4 × 1] *k*-point grid was adopted. ACID calculations were carried out using the AICD 3.0.4 program,^36^ and NICS(1) values were computed with CP2K.^48^

In-solution synthesis of **755-Prec** was carried out following an already described protocol^49^ (see section S16 in Supporting Information).

## Supplementary Material



## Notes

Self-assembly of **755-Prec**; length distribution analysis of **755-Poly**; relative abundance of *cis* and *trans* configurations; correlation factor calculation; dipole moments of related [7–5–5] configurations; extended STS spectra; extended set of d*I*/d*V* maps; comparison of the electronic properties of the *cis* and *trans* configurations; effect of the herringbone reconstruction on the d*I*/d*V* maps; splitting of the heptagon region at high positive bias voltages; DFT-calculated HOMO and LUMO of an isolated molecular unit; effect of polymerization on the bandgap evolution; evolution of the HOMO–LUMO gaps; NICS(1) aromatic comparison between benzene, azulene, pentalene, and the molecular unit of **755-Poly**; extended theoretical methods; and in-solution synthesis of **755-Prec** (PDF)
