# Supplementary material for: On-Surface Synthesis of a Low Bandgap Cyclopenta[c,d]azulene-Based Polymer
Source: J Am Chem Soc. 2026 Oct 14;148(42):45419–25. doi: 10.1021/jacs.6c08825 (PMC13721034; doi:10.1021/jacs.6c08825)
Supplement: Supplementary file 1 [file ja6c08825_si_001.pdf]

On-Surface Synthesis of a Low Bandgap Cyclopenta[*c,d*]azulene-Based Polymer

S. Jiménez-Fernández[1†], F. Villalobos[2†], V. Kumar[3], J. I. Martínez[1], J. I. Mendieta-Moreno[4], P. L. de Andrés[1], E. Tosi[1], J. A. Martín-Gago[1], J. M. Cuerva[3], I. Palacio[1], A. G. Campaña[3*], C. Sánchez-Sánchez[1*]

[1] ESISNA Group, Instituto de Ciencia de Materiales de Madrid (ICMM-CSIC), Sor Juana Inés de la Cruz 3, 28049, Madrid (Spain)

[2] Institute of Physics of the Czech Academy of Sciences, Cukrovarnická 112/10, 162 00, Prague 6 (Czech Republic)

[3] Departamento de Química Orgánica, Universidad de Granada (UGR), Unidad de Excelencia de Química UEQ, C. U. Fuentenueva, 18071, Granada (Spain)

[4] Departamento de Física Teórica de la Materia Condensada, Universidad Autónoma de Madrid, 28049, Madrid (Spain)

E-mail: araceligc@ugr.es, cssanchez@icmm.csic.es

Table of Contents

1. Self-Assembly of **755-Prec**. 3

2. Length Distribution Analysis of **755-Poly**. 4

3. Relative Abundance of *Cis* and *Trans* Configurations. 5

4. Correlation Factor Calculation. 6

5. Dipole Moments of Related [7-5-5] Configurations. 7

6. Extended STS Spectra. 8

7. Extended Set of dI/dV Maps. 9

8. Comparison of the Electronic Properties of the *Cis* and *Trans* Configurations. 10

9. Effect of the Herringbone Reconstruction on the dI/dV Maps. 12

10. Splitting of the Heptagon Region at High Positive Bias Voltages. 13

11. DFT-Calculated HOMO and LUMO of an Isolated Molecular Unit. 14

12. Effect of Polymerization on the Bandgap Evolution. 15

13. Evolution of the HOMO-LUMO Gaps. 16

14. NICS(1) Aromatic Comparison Between Benzene, Azulene, Pentalene, and the Molecular Unit of **755-Poly**. 18

15. Extended Theoretical Methods. 19

16. In-solution Synthesis of **755-Prec**. 20

References 24

1. Self-Assembly of **755-Prec**.

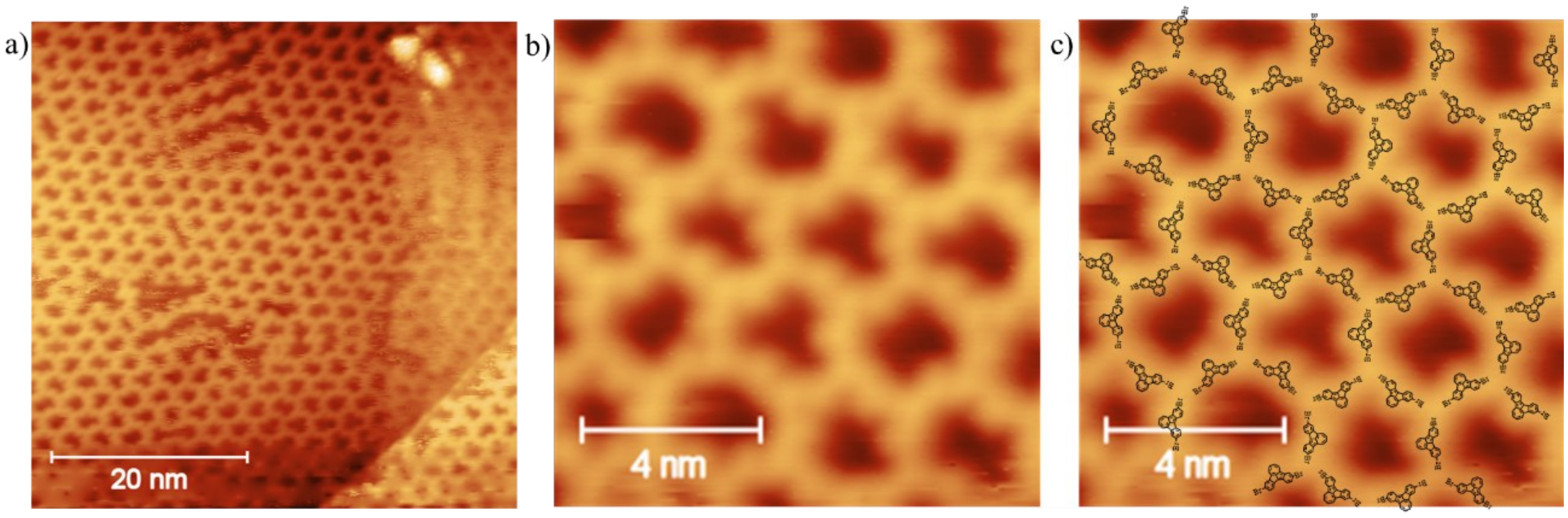

Figure S1. a) and b) Topographic STM images of the surface after 5 min of evaporation with the substrate at room temperature, where the self-assembly is observed. c) Same image of b), but with the precursor scheme superimposed. Images taken at LN_2 temperature. V_{bias} = 1200 mV; I_t = 20 pA.

Figure S1 shows the surface after 5 min of evaporation with the substrate at RT, where the self-assembled phase is observed. The self-assembly is stabilized by trimers of Br atoms. No preferential distribution of the *cis* and *trans* configurations along the self-assembly phase is observed. Importantly, there is no preferential distribution in the number of heptagons oriented toward the pore, finding pores containing from one to four heptagons.

2. Length Distribution Analysis of **755-Poly**.

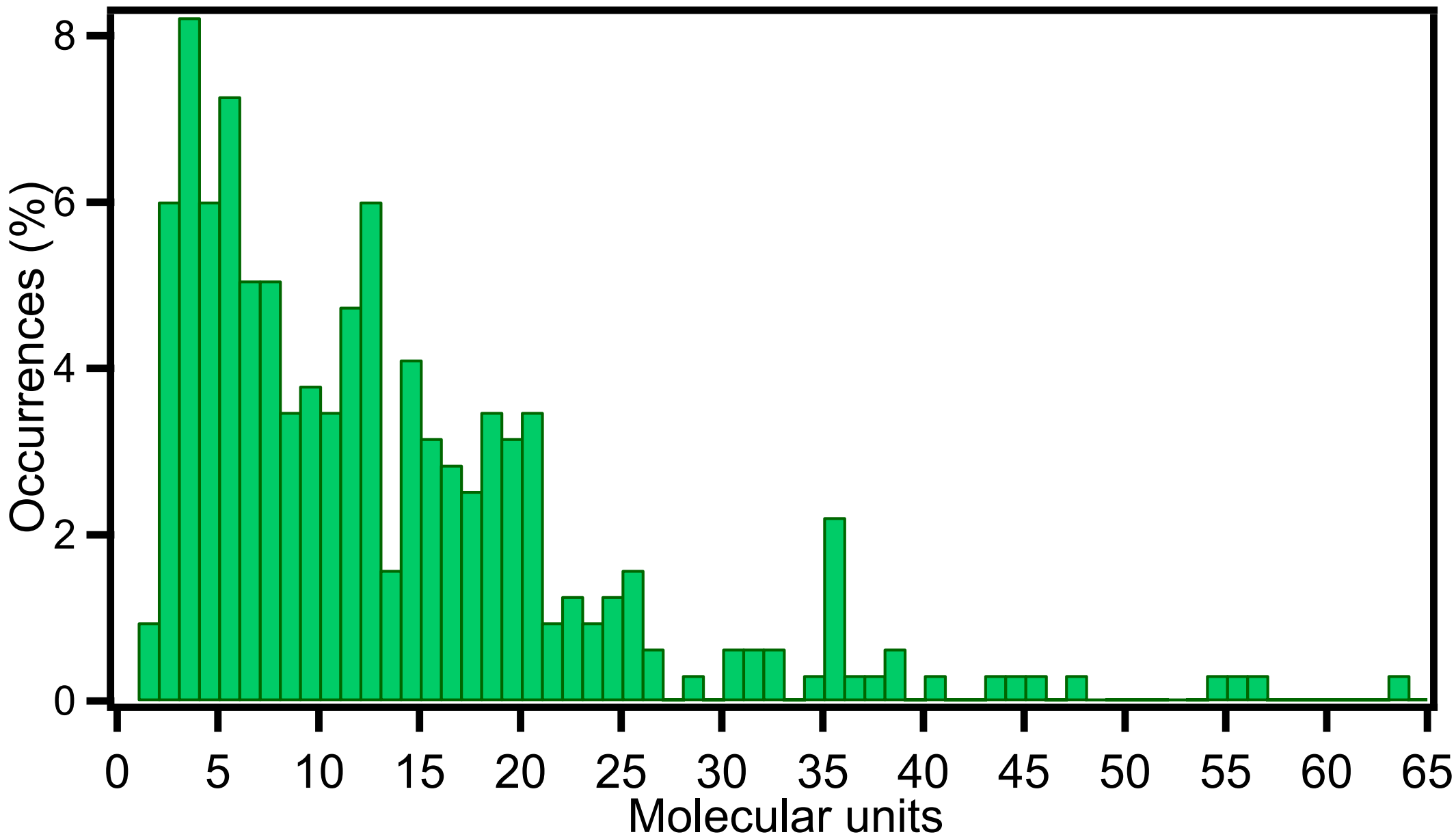

Figure S2. The histogram shows the statistical distribution of the polymer length. This analysis has been performed on high-resolution STM images obtained with a CO-functionalized tip, covering more than 300 polymers.

Figure S2 shows the length distribution of the polymers, evidencing polymers exceeding 60 molecular units within the distribution.

3. Relative Abundance of *Cis* and *Trans* Configurations.

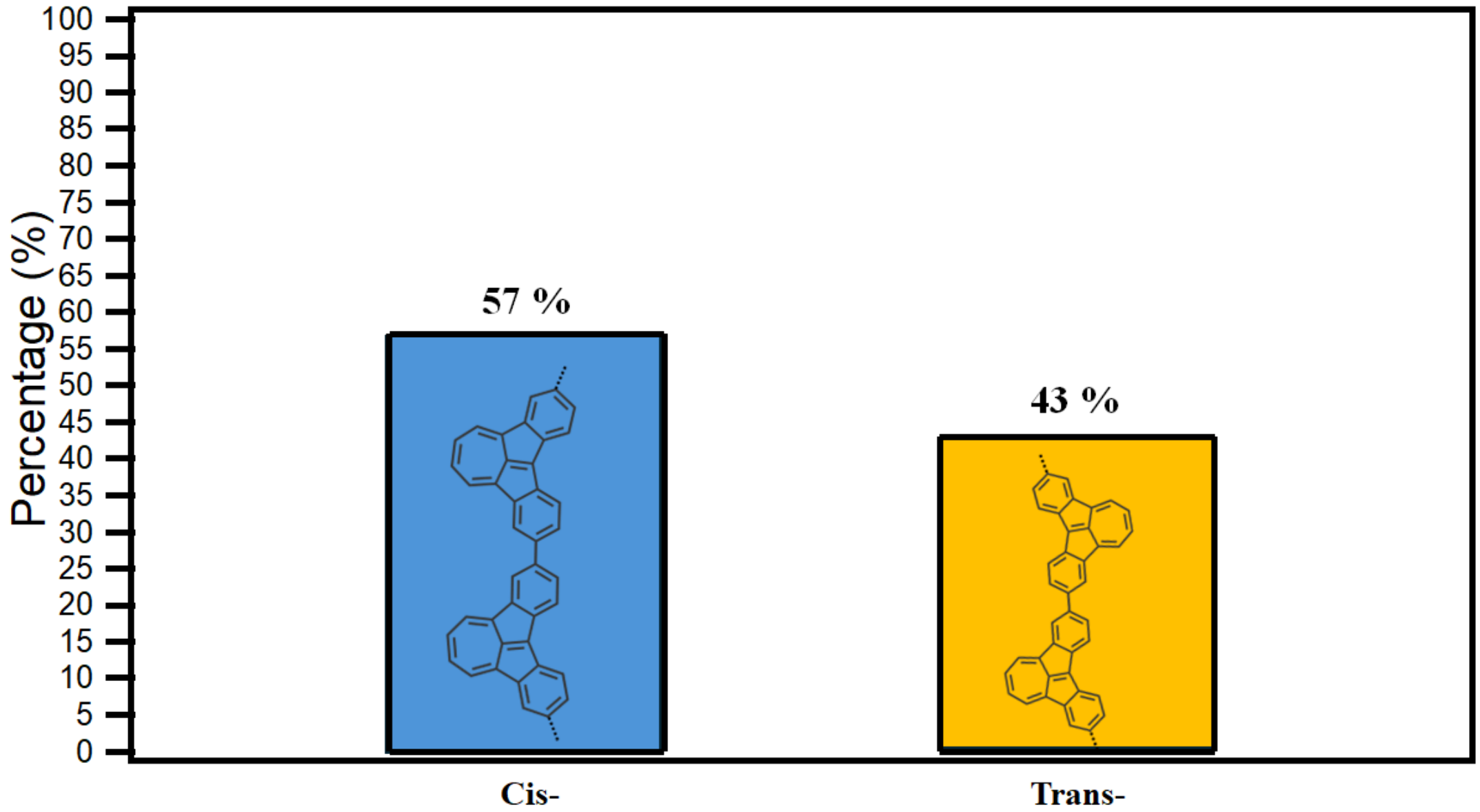

Figure S3. The histogram displays the statistical distribution of cis- and trans-configurations. This analysis has been performed on high-resolution STM images obtained with a CO-functionalized tip, covering ~1500 molecular units.

Figure S3 shows the histogram showing the statistical distribution of *cis* and *trans* configurations. This analysis has been performed on high-resolution STM images obtained with a CO-functionalized tip, covering ~1500 molecular units.

4. Correlation Factor Calculation.

The correlation factor analysis has been carried out over more than 1500 molecular units arranged along different polymers. Correlation factors have been calculated using the Pearson correlation coefficient with numpy package. For each polymer, a dichotomous variable for the *cis* or *trans* configurations (coded as 0 and 1) has been assigned and the Pearson's correlation coefficient was calculated between each value in the series and its immediately following value, yielding a lag-1 autocorrelation coefficient.

Pearson's correlation coefficient was calculated as:

$$r = \frac{\sum_{t=1}^{n} (x_t - \bar{x})(x_{t+1} - \bar{x}_{+1})}{\sqrt{[\sum_{t=1}^{n} (x_t - \bar{x})^2]} \cdot \sqrt{[\sum_{t=1}^{n} (x_{t+1} - \bar{x}_{+1})^2]}}$$

Equation 1.

where: x_t = value of the observation at time t, x_{t+1} = value of the immediately following observation, $\bar{x}$ = mean of the series, $\bar{x}_{+1}$ = mean of the lagged series.

5. Dipole Moments of Related [7-5-5] Configurations.

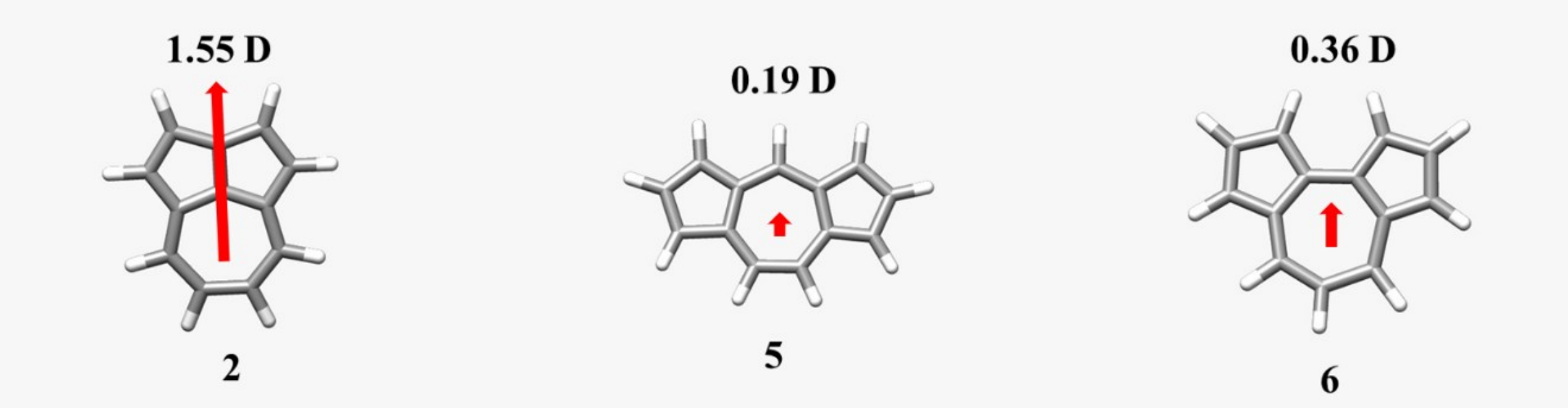

*Figure S4. DFT calculations of the dipole moments of the configurations **2**, **5** and **6**. The red arrows indicate the direction of the dipole moment with their length being proportional to the total dipole moment.*

Figure S4 shows the net dipole moment values for configurations **2**, **5** and **6**. The large dipole moment in configuration **2** arises from the charge redistribution among neighboring atoms within the seven-membered and the adjacent five-membered rings, which aligns the polarization along a common direction. In contrast, for configurations **5** and **6**, charge donation to both flanking five-membered rings produces nearly antiparallel polarization vectors that largely cancel, yielding small net dipole moments of 0.19 D and 0.36 D, respectively.

6. Extended STS Spectra.

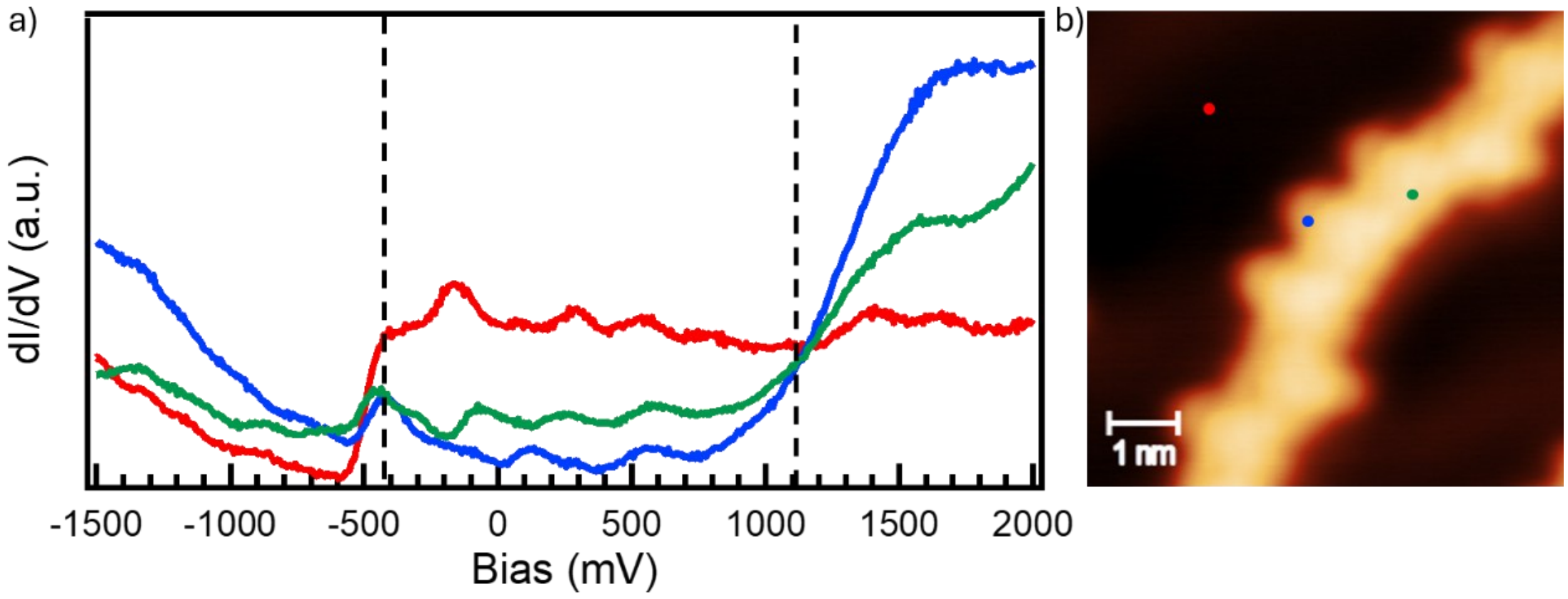

Figure S5. a) Extended STS spectra plotted on the same y-scale. The exact points at which the spectra were acquired are indicated by the color-coded circles in the STM image (b). V_{bias} = 300 mV. I_t = 100 pA.

7. Extended Set of dI/dV Maps.

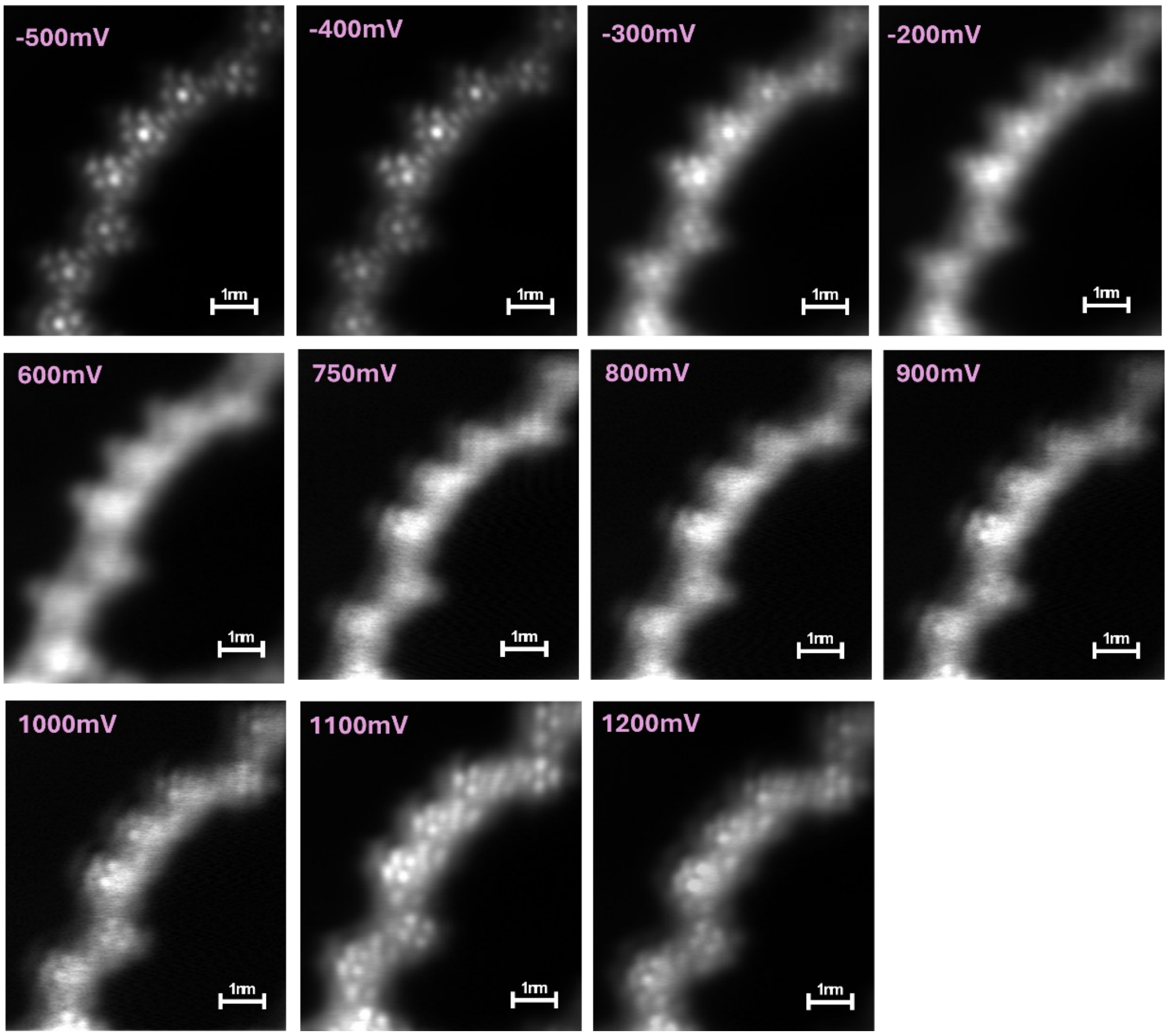

Figure S6. Series of dI/dV maps acquired at the bias voltages indicated in purple. Lock-in parameters: V_{ac} = 50 mV, f = 769 Hz.

Figure S6 shows the dI/dV maps acquired at the bias voltages indicated in purple. These maps support the assignment of the HOMO and LUMO, which was based on the identification of spectral features whose corresponding dI/dV maps most clearly resolve the relevant spatial pattern, namely at -400 mV and 1100 mV.

8. Comparison of the Electronic Properties of the *Cis* and *Trans* Configurations.

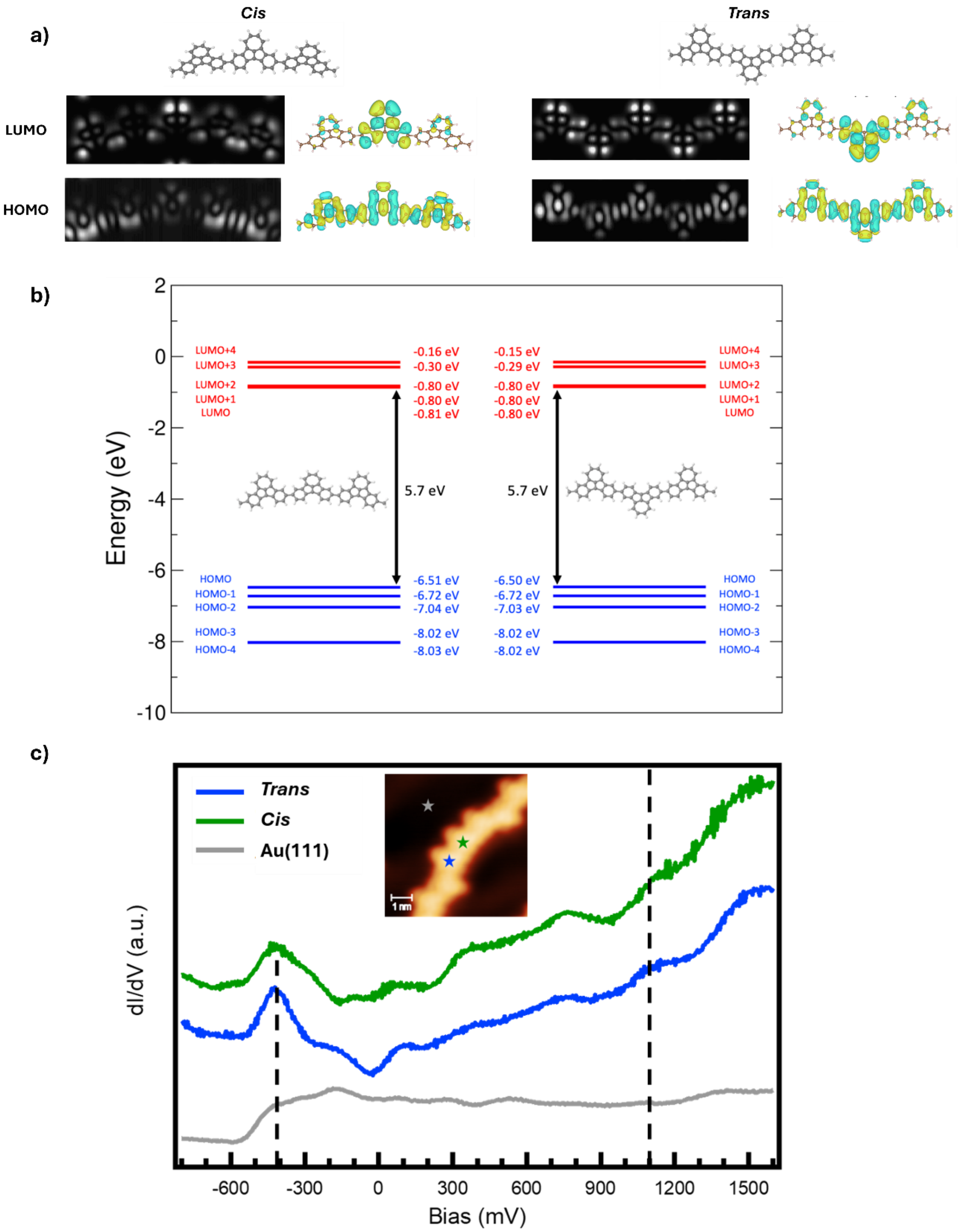

Figure S7. a) DFT calculations of the HOMO and LUMO molecular orbitals for configurations in a 3-unit oligomer, and dI/dV maps for a 5-unit oligomer (both oligomers being terminated by methyl groups). There are no substantial differences between both configurations. b) Band diagrams for cis *and* trans *polymers. c) STS spectra, with the acquisition positions color-coded in the STM image shown in the inset.* V_{bias} *= 300 mV.* I_t *= 100 pA.*

Figure S7 presents the comparison of the electronic properties of the *cis* and *trans* polymers. Figure S7a shows the DFT calculations of the spatial distribution of the HOMO and LUMO. We observe that the features hardly vary between *cis* and *trans* configurations. This is further

corroborated by the theoretical molecular orbitals (Figure S7a) and band diagrams (Figure S7b), which exhibit identical features in both cases. Figure S7c shows experimental evidence for the lack of differences between both configurations by means of STS. The green curve corresponds to a molecular unit in purely *cis* configuration while the blue one is for a molecular unit in *cis* and *trans* configurations. As it can be observed, they display essentially identical electronic features. Although the blue spectrum does not correspond to a complete molecular unit connected exclusively through *trans* configurations, any significant difference in the electronic properties associated with *cis* and *trans* configurations would be reflected in a comparison between these two spectra. This is not the case, as both curves show the HOMO and LUMO at −400 mV and 1100 mV, respectively, as indicated by the black dashed lines.

9. Effect of the Herringbone Reconstruction on the dI/dV Maps.

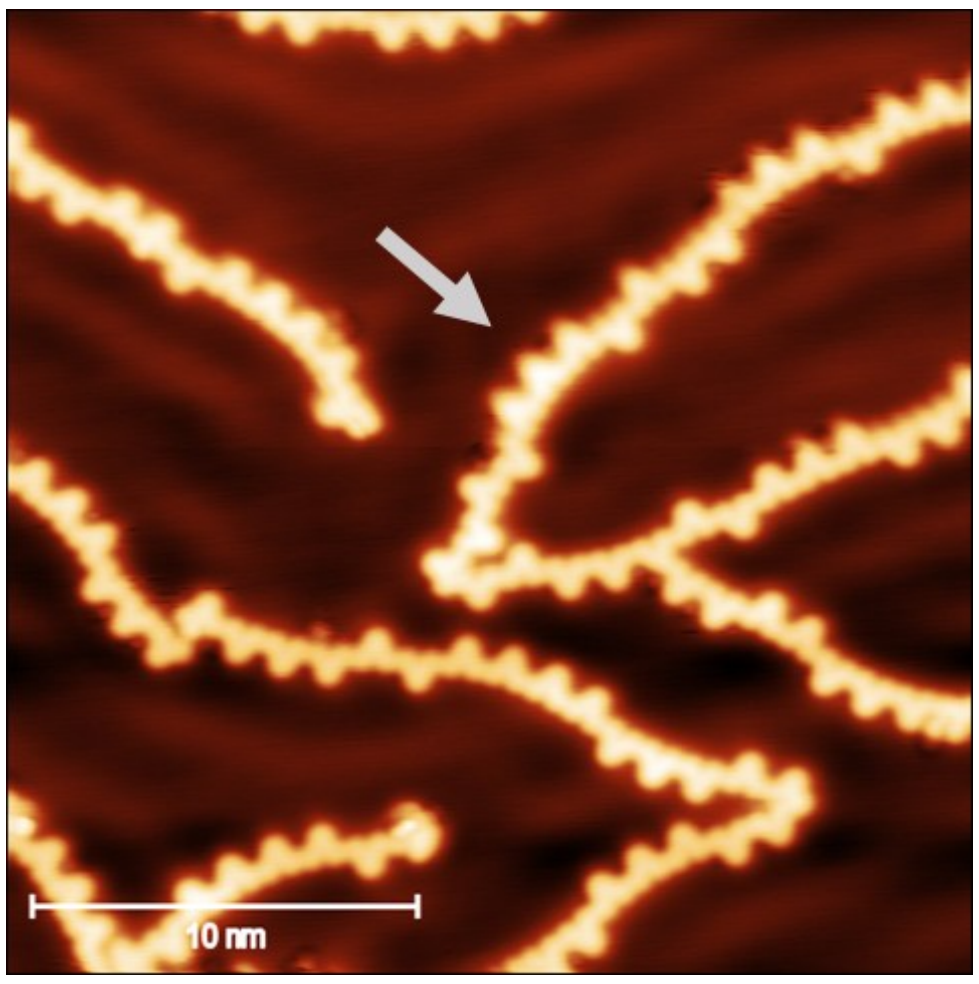

Figure S8. Overview topographic STM image showing the polymer where the dI/dV maps presented in the main text were measured, marked by a grey arrow. The herringbone reconstruction of the Au(111) substrate crossing the polymer is also visible, which leads to a higher contrast of the molecular units in the constant-height dI/dV maps. V_{bias} *= 130 mV;* I_t *= 100 pA.*

Figure S8 shows an overview STM image where the polymer segment used in the dI/dV maps is marked by the grey arrow. It can be clearly discerned how the herringbone reconstruction crosses the polymer at the position where a higher contrast is observed in the constant-height dI/dV maps.

10. Splitting of the Heptagon Region at High Positive Bias Voltages.

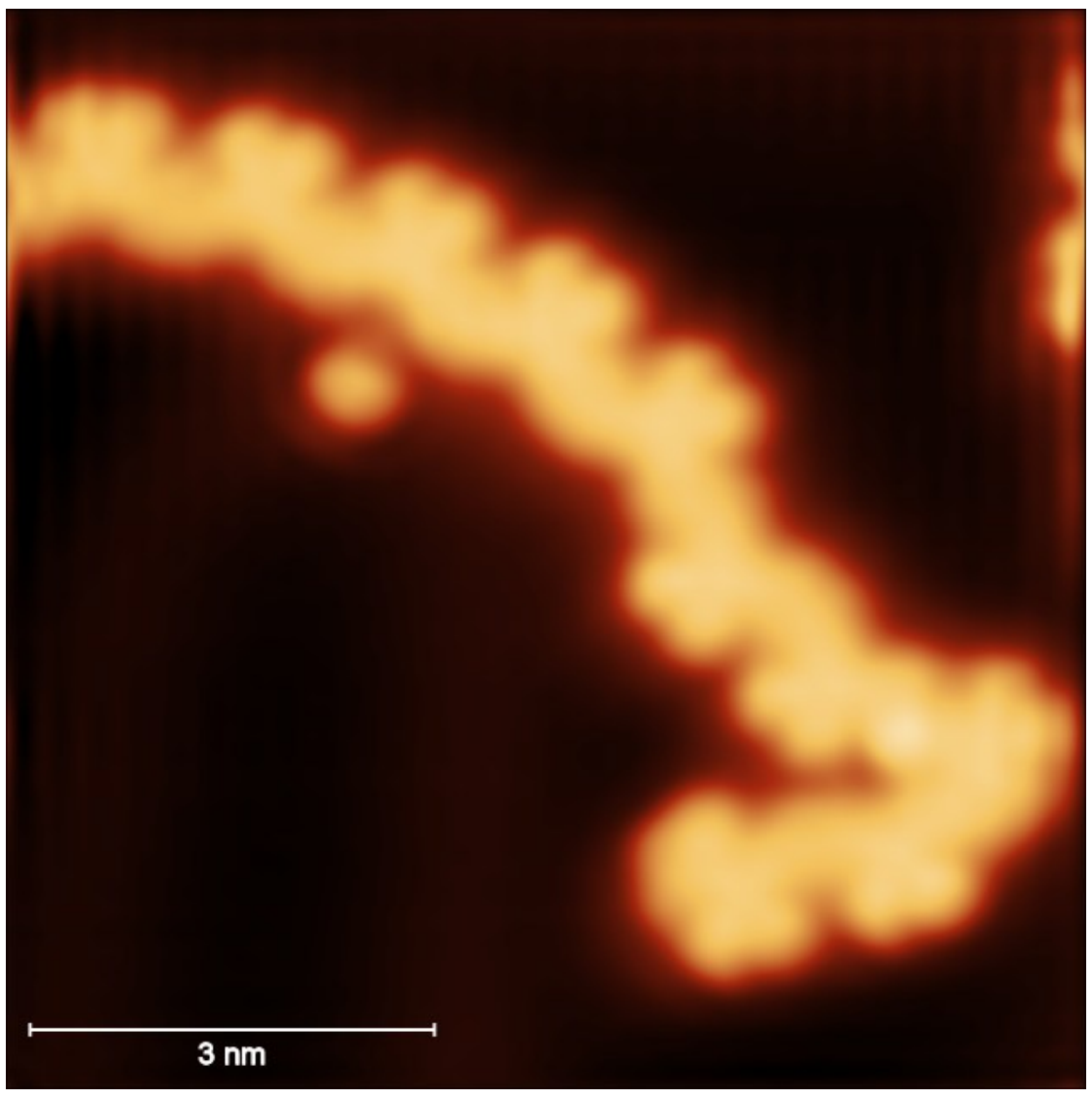

Figure S9. Overview topographic STM image showing the polymer where the dI/dV maps presented in the main text were measured, marked by a grey arrow. The herringbone reconstruction of the Au(111) substrate crossing the polymer is also visible, which leads to a higher contrast of the molecular units in the constant-height dI/dV maps. V_{bias} *= 1600 mV;* I_t *= 50 pA.*

Figure S9 shows the splitting of the heptagon region into two lobes at high positive bias voltages, thereby mimicking the LUMO features discussed in the manuscript.

11. DFT-Calculated HOMO and LUMO of an Isolated Molecular Unit.

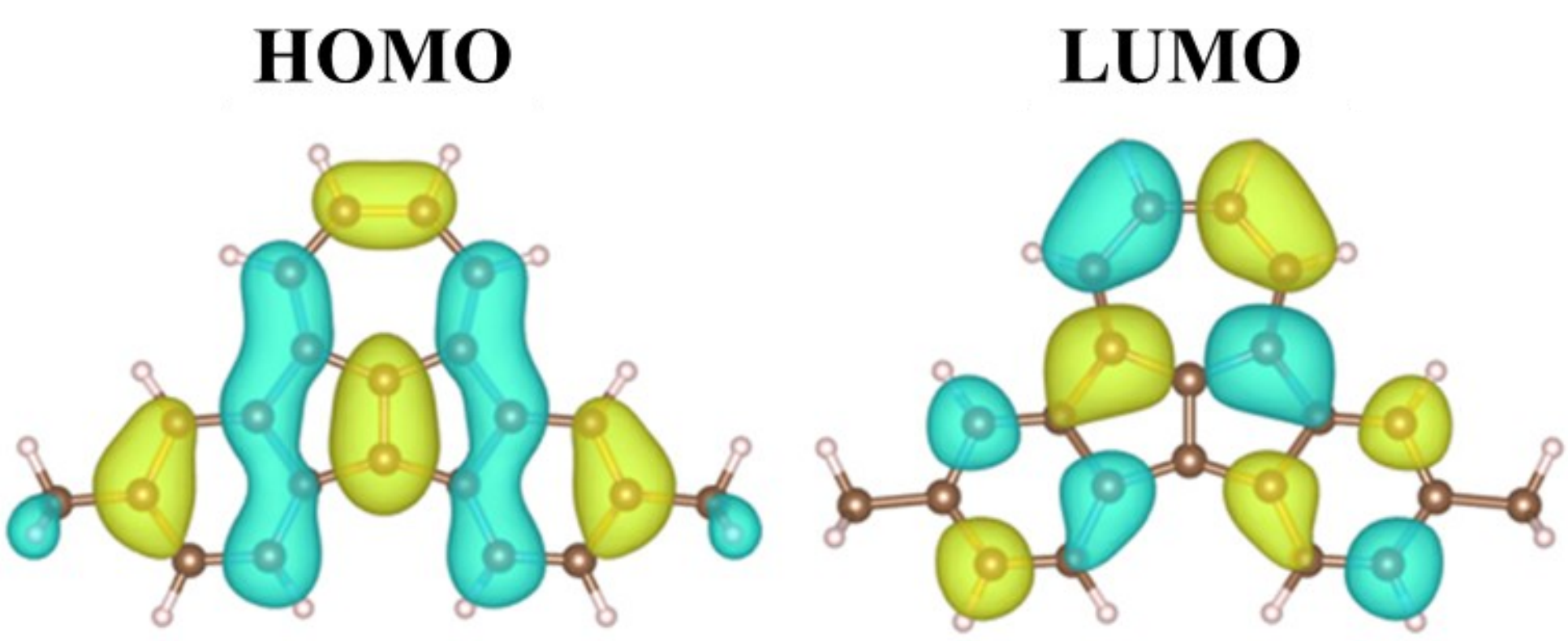

Figure S10. Calculations of the spatial distribution of the HOMO and the LUMO of a methyl-terminated molecular unit in gas phase.

Figure S10 shows the calculated spatial distributions of the HOMO and LUMO of a methyl-terminated molecular unit in the gas phase. These features match the dI/dV maps in the main text, where it is explained that this similarity arises from the fact that the molecular units forming the polymer are connected by only a single bond, which implies the reduction of π-conjugation.

12. Effect of Polymerization on the Bandgap Evolution.

To address the effect of polymerization on the evolution of the bandgap, we have performed an extensive series of high-accuracy molecular electronic structure calculations on isolated gas-phase oligomers containing from one to six repeating units (Figure S11). These calculations were carried out using the range-separated hybrid functional ωB97X-D, one of the most accurate density functionals currently available in Gaussian for predicting molecular frontier orbital energies and electronic bandgaps. In addition to the conventional HOMO-LUMO orbital gaps, we also computed the fundamental gaps using the ΔSCF approach, which is generally regarded as a considerably more reliable approximation to the molecular quasiparticle gap than the Kohn–Sham orbital gap alone. Consequently, this set of calculations provides a rigorous description of the intrinsic electronic evolution of the isolated polymer fragments, independent of substrate effects or the well-known bandgap underestimation associated with semilocal DFT.

The calculations reveal that the HOMO-LUMO gap decreases only moderately (~7.5 %) from 6.00 eV for the monomer to 5.55 eV for the six-units oligomer, while the ΔSCF gap decreases from 5.59 eV to 4.98 eV over the same series (~11.0 %). Moreover, we investigated different polymer configurations, including fully *cis* sequences and alternating *trans* arrangements. Remarkably, both structural motifs yield virtually identical electronic bandgaps, demonstrating that the electronic structure is essentially insensitive to the local *cis/trans* connectivity pattern. Most importantly, both the orbital and ΔSCF bandgaps rapidly converge with increasing oligomer length, indicating that polymerization itself produces only a limited additional reduction of the intrinsic molecular gap. This behavior is fully consistent with the structural characteristics of **755-Poly**, where neighboring units are connected through single-like C—C bonds, resulting in a relatively weak π-electronic communication along the backbone. Consequently, the electronic structure of the polymer remains largely that of the individual [7–5–5] building block.

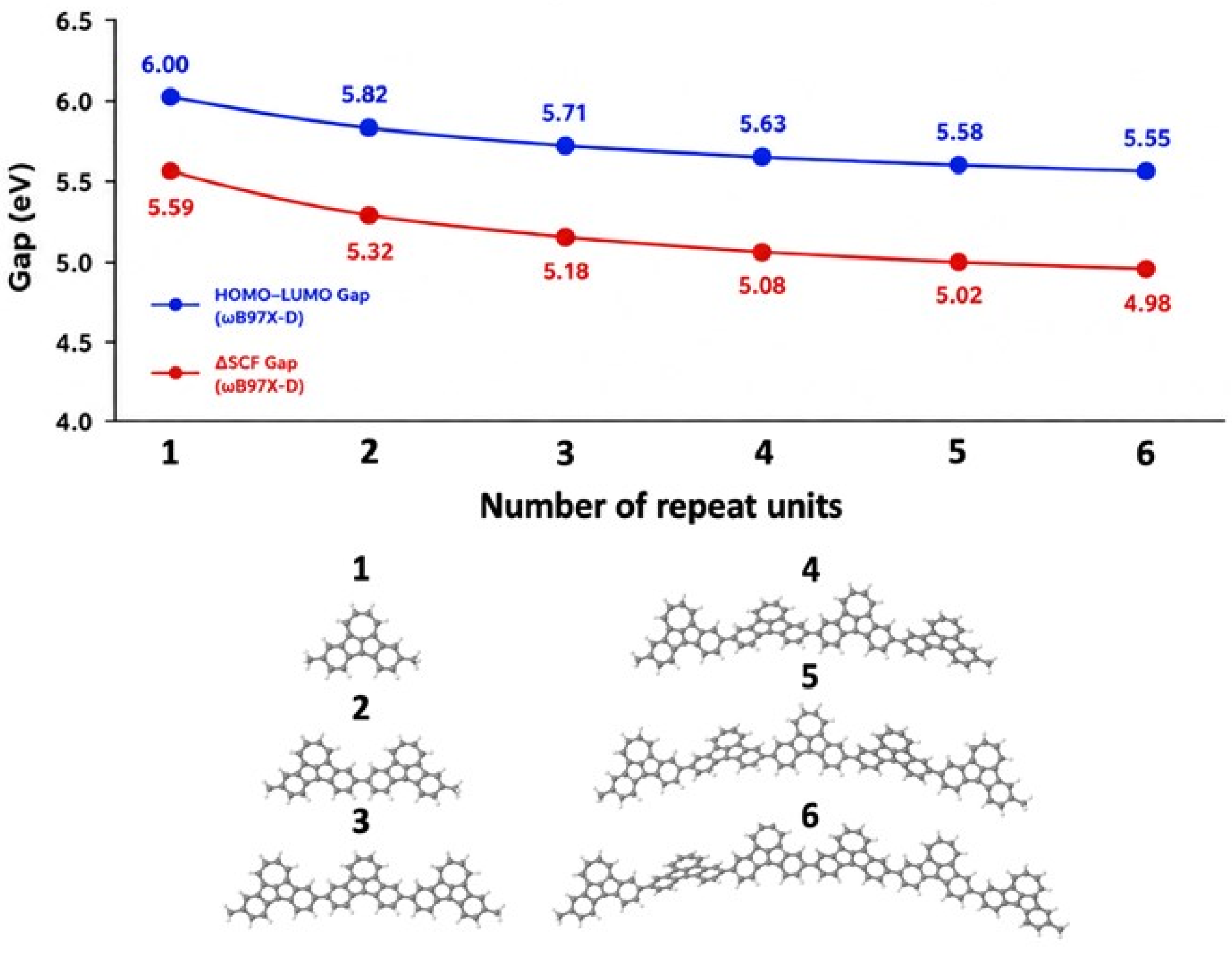

Figure S11. Evolution of the electronic bandgap with oligomer length (gas-phase calculations).

13. Evolution of the HOMO-LUMO Gaps.

To determine the effect in the bandgap of the polymer length and its adsorption on the Au(111) surface, we have computed the electronic gaps for the isolated monomer, a 3-unit representative oligomer, the infinite free-standing polymer (in a cis-trans-cis-trans… fashion to facilitate the commensurability with the substrate in the periodic calculations of the polymer on Au), and the polymer adsorbed on Au(111) (Figure S12).

Importantly, to ensure that the comparison reflects genuine physical trends rather than methodological differences, all these four systems were evaluated using exactly the same periodic DFT framework employed throughout the manuscript, namely GGA-PBESol+D3. It is important to note that these calculations are different and have a different purpose to those presented in section 12. Those were specifically designed to assess the intrinsic effect of polymerization on finite molecular fragments and provide a much better approximation to the molecular fundamental (quasiparticle) bandgap. However, the corresponding values cannot be directly compared with periodic Kohn-Sham bandgaps, since they represent different physical quantities. On the other hand, these calculations allow investigating the electronic properties of periodic systems adsorbed on a surface, something unaffordable within the previous computational framework.

Our results indicate that the isolated monomer exhibits a Kohn-Sham HOMO-LUMO bandgap of 1.30 eV. The gap decreases to 1.01 eV for the trimer and reaches 0.97 eV for the infinite free-standing polymer calculated using periodic boundary conditions and a 6×1×1 Monkhorst–Pack k-point sampling. This evolution demonstrates that the electronic structure converges rapidly with increasing oligomer length, in excellent agreement with the independent ωB97X-D/ΔSCF calculations presented above.

When the polymer is adsorbed on the Au(111), our analysis of the ribbon-projected electronic band structure yields a gap of 0.94 eV. Therefore, adsorption on the metallic substrate produces only a very small renormalization (around 0.03 eV) of the intrinsic polymer gap. Although the complete polymer/Au(111) system naturally contains metallic Au states crossing the Fermi level, the projected electronic states of the polymer itself preserve essentially the same gap as in the free-standing periodic ribbon, indicating that the interaction with Au(111) is dominated by weak physisorption and induces only minor perturbations of the intrinsic electronic structure.

The two complementary computational approaches employed in this work lead to the same physical conclusion. The high-level molecular calculations demonstrate that extending the conjugation from the monomer to long oligomers produces only a modest reduction of the intrinsic molecular gap, while the periodic DFT calculations show that this electronic structure is already essentially converged at the polymer limit and remains almost unchanged after adsorption on Au(111). Consequently, the experimentally observed narrow-bandgap character of **755-Poly** should not be interpreted as a consequence of long-range polymer conjugation or substrate-induced electronic renormalization. Instead, it primarily originates from the intrinsic electronic structure of the cyclopenta[c,d]azulene [7-5-5] building block, whose non-alternant topology and associated intramolecular charge redistribution determine the low-bandgap electronic character that is subsequently preserved upon polymerization and adsorption.

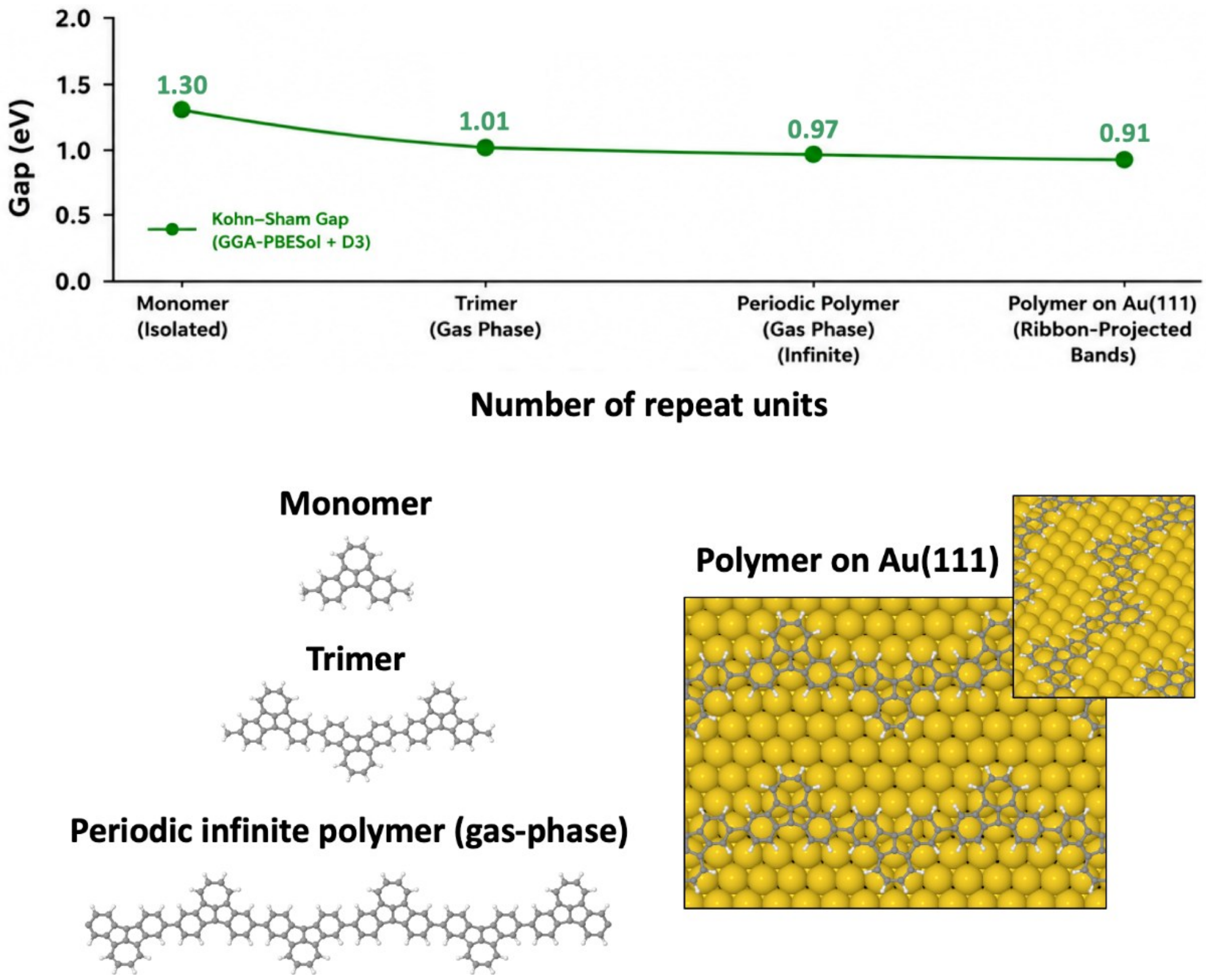

Figure S12. Evolution of the calculated electronic bandgap from the isolated monomer to the Au(111)-supported polymer, showing the rapid convergence of the Kohn-Sham bandgap with increasing oligomer length and its negligible renormalization upon adsorption on the metallic substrate.

14. NICS(1) Aromatic Comparison Between Benzene, Azulene, Pentalene, and the Molecular Unit of **755-Poly**.

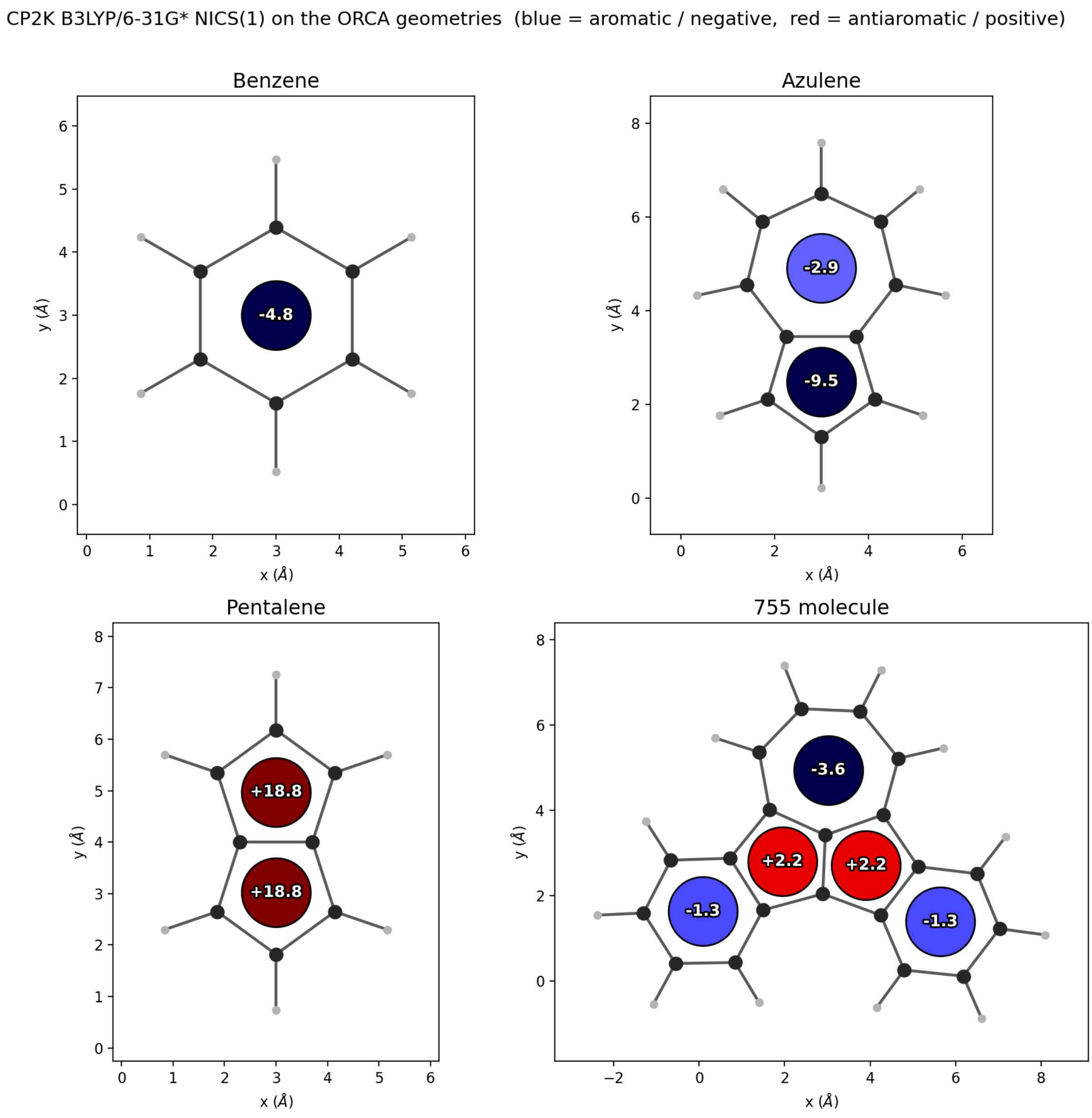

*Figure S13. NICS(1) calculations of benzene, azulene, pentalene, and one unit of **755-Poly**.*

15. Extended Theoretical Methods.

The charge redistribution shown in Figure 4c was obtained from Hirshfeld population analysis performed with the CP2K package. Likewise, the NICS(1) calculations were carried out using CP2K on fully relaxed gas-phase geometries. Both the geometry optimization and the subsequent NICS calculations were performed at the B3LYP/6-31G* level, ensuring complete methodological consistency between the optimized structures and the magnetic shielding calculations.

The magnetic response method used in CP2K has been compared with other theoretical methods and already validated.[1]

The periodic calculations were performed using Quantum ESPRESSO with the PBESol exchange-correlation functional, Grimme D3 dispersion corrections, and RRKJ ultrasoft pseudopotentials. A kinetic-energy cutoff of 40 Ry was employed for the wavefunctions and 300 Ry for the charge density after convergence testing. The Au(111) substrate was modeled using a four-layer slab with the bottom two layers kept fixed during structural relaxation. A vacuum region larger than 20 Å was introduced to avoid interactions between periodic images, and Brillouin-zone integrations were performed using a 1 × 4 × 1 Monkhorst–Pack k-point mesh.

The supported polymer was represented by a methyl-terminated six-unit oligomer adsorbed on Au(111). This choice was motivated not only by computational efficiency but also by the electronic convergence of the system.

The bond-resolved STM images were simulated using the Probe Particle (PP)-STM method with a CO-functionalized tip, employing the relaxed periodic DFT geometries and associated electronic densities as input.

Gaussian16 (ωB97X-D and B3LYP/6-31G**) was employed for the high-accuracy molecular calculations on the isolated oligomers and for the molecular geometry optimizations. CP2K (B3LYP/6-31G*) was used for the Hirshfeld charge analysis and the NICS(1) calculations. Quantum ESPRESSO (PBESol+D3) was employed for all periodic calculations, including the free-standing polymer, the polymer adsorbed on Au(111), relaxed adsorption geometries, projected band structures, and electronic densities, and iv) PP-STM was used for the simulation of the bond-resolved STM images.

16. In-solution Synthesis of **755-Prec**.

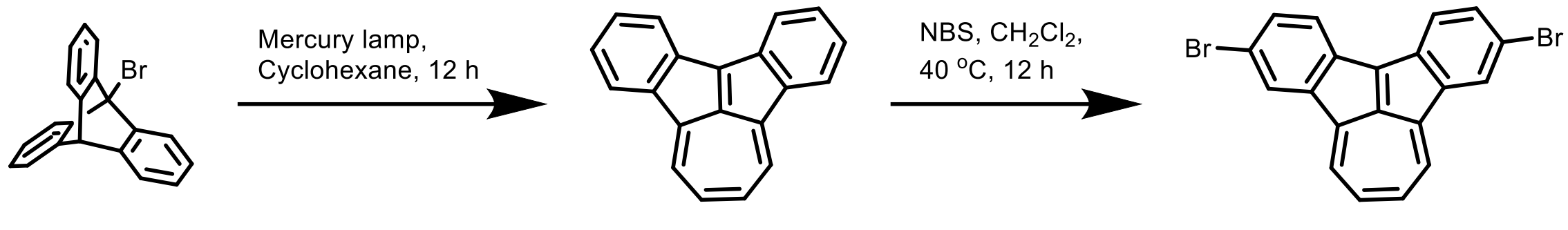

*Scheme 1. In-solution synthesis route for **755-Prec**.*

Synthesis of Benz[*a*]indeno[1,2,3-*cd*]azulene (1). The synthesis was carried out according to a reported procedure[2]. A solution of 9-bromo triptycene (3.2 mmol) in cyclohexane (1 L) was degassed by bubbling argon through the solution for 1 h at room temperature. Triethylamine (0.5 ml) was added and the solution was irradiated under an argon atmosphere in a quartz vessel with a low-pressure mercury lamp for 12 h. The solvent was removed under reduced pressure and the residue was purified by column chromatography on silica gel (eluent: hexane). The fraction corresponding to the green band was collected and concentrated to afford compound **1** as a brown solid (23% yield).

^{1}H NMR (400 MHz, CDCl3): **δ [ppm]** = 8.48 (dd, *J*=5.5, 2 H), 8.35 (d, *J*=7.7 Hz, 2 H), 8.07 (d, *J*=7.6 Hz, 2 H), 7.93 (dd, *J*=5.6, 2 H), 7.70 (t, *J*=7.5 Hz, 2 H), 7.42 ppm (t, *J*=7.5 Hz, 2 H)

Synthesis of 755-Prec. The reaction was carried out according to a reported procedure with minor modifications in the equivalents of N-bromosuccinimide.[1] A solution of **1** (30 mg, 0.11 mmol) and N-bromosuccinimide (33 mg, 0.19 mmol) in dichloromethane (10 mL) was heated at 40 °C for 12 h. After completion of the reaction, the solvent was removed under reduced pressure and the residue was purified by column chromatography on silica gel using hexane as the eluent. The reaction afforded the desired dibrominated compound **755-Prec** in 43% yield as dark brown solid.

^{1}H NMR (400 MHz, CDCl3): **δ [ppm]** = 8.42 (dd, *J*=5.6, 2 H), 8.39 (s, 2 H), 7.95 (dd, *J*=5.6, 2 H), 7.82 (d, *J*=7.7 Hz, 2 H),7.76 ppm (d, *J*=7.7 Hz, 2 H)

^{13}C NMR (126 MHz, $CDCl_3$): **δ [ppm]** = 139.7, 139.6, 138.1, 132.6, 131.7, 125.9, 124.5, 121.0, 116.5 ppm

HRMS (ESI/Q-TOF) m/z: $[M]^+$ Calculated for $C_{20}H_{10}Br_2$ 407.9149; Found 407.9164.

NMR Spectra:

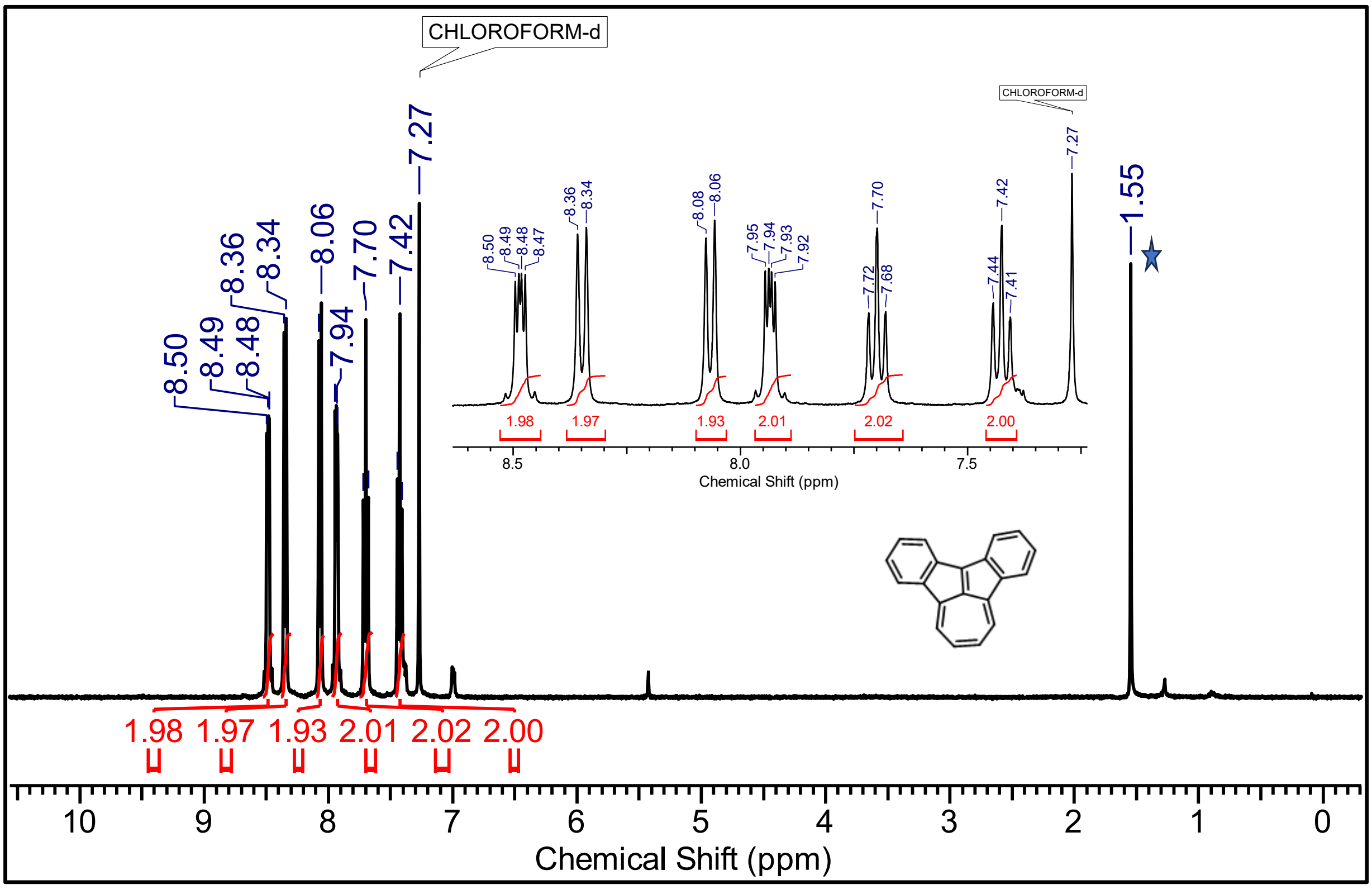

Figure S14. ^{1}H NMR (400 MHz) spectrum of 1 in $CDCl_3$ at 298 K (: Water).*

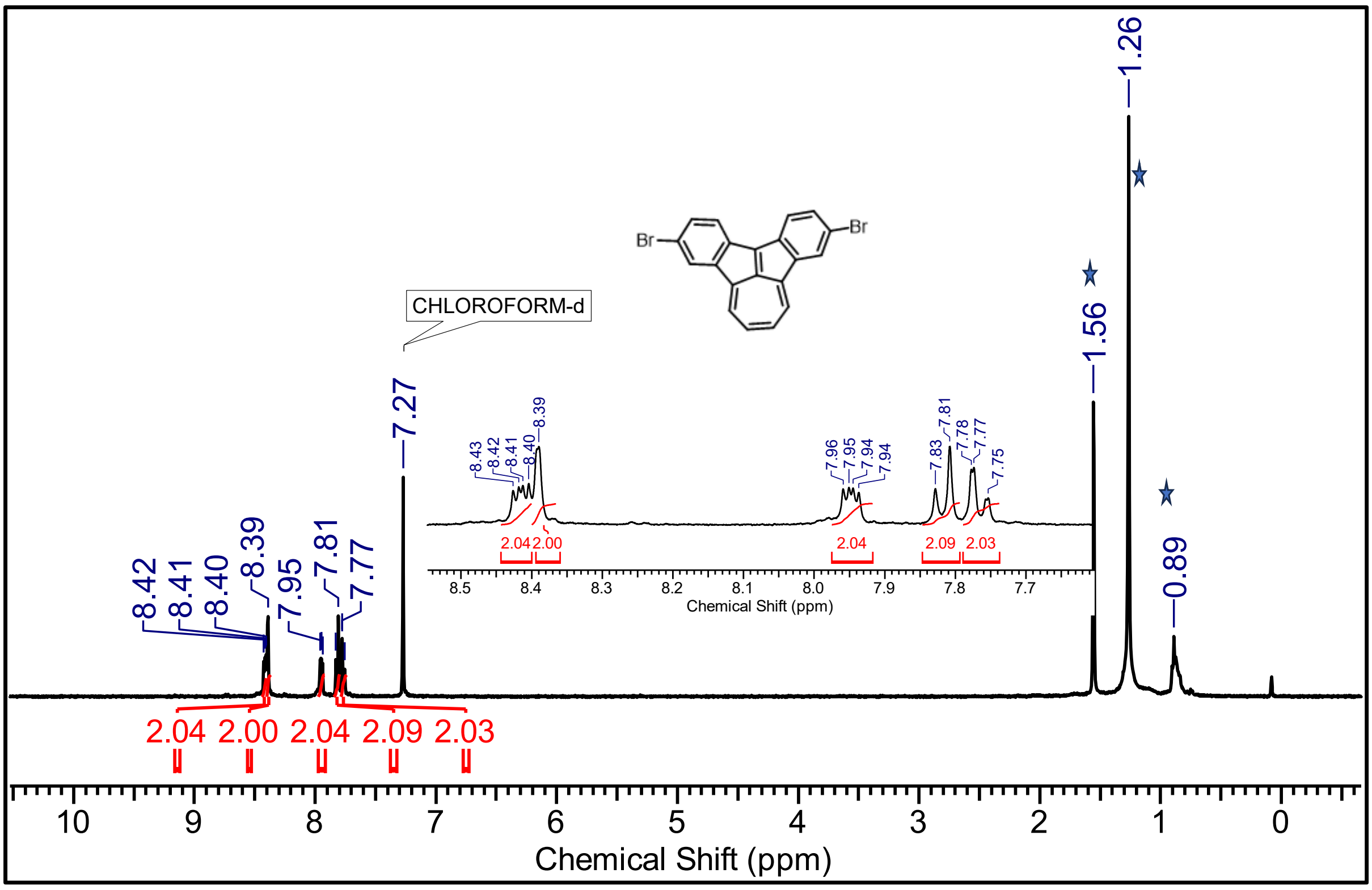

*Figure S15. ^{1}H NMR (400 MHz) spectrum of **755-Prec** in $CDCl_3$ at 298 K (*: Water and grease impurity).*

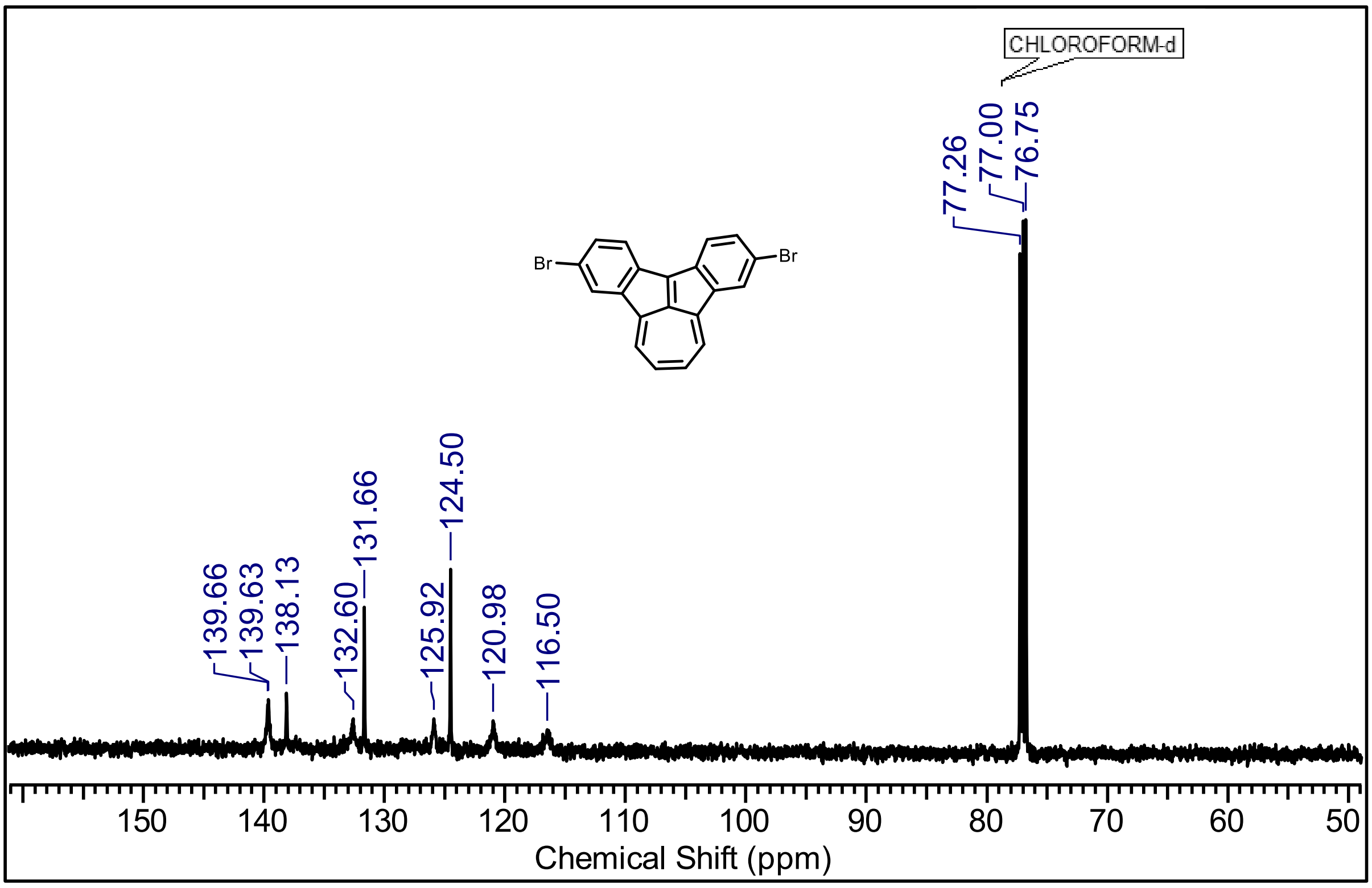

*Figure S16. ^{13}C NMR (126 MHz) spectrum of **755-Prec** in $CDCl_3$ at 298 K.*

HRMS:

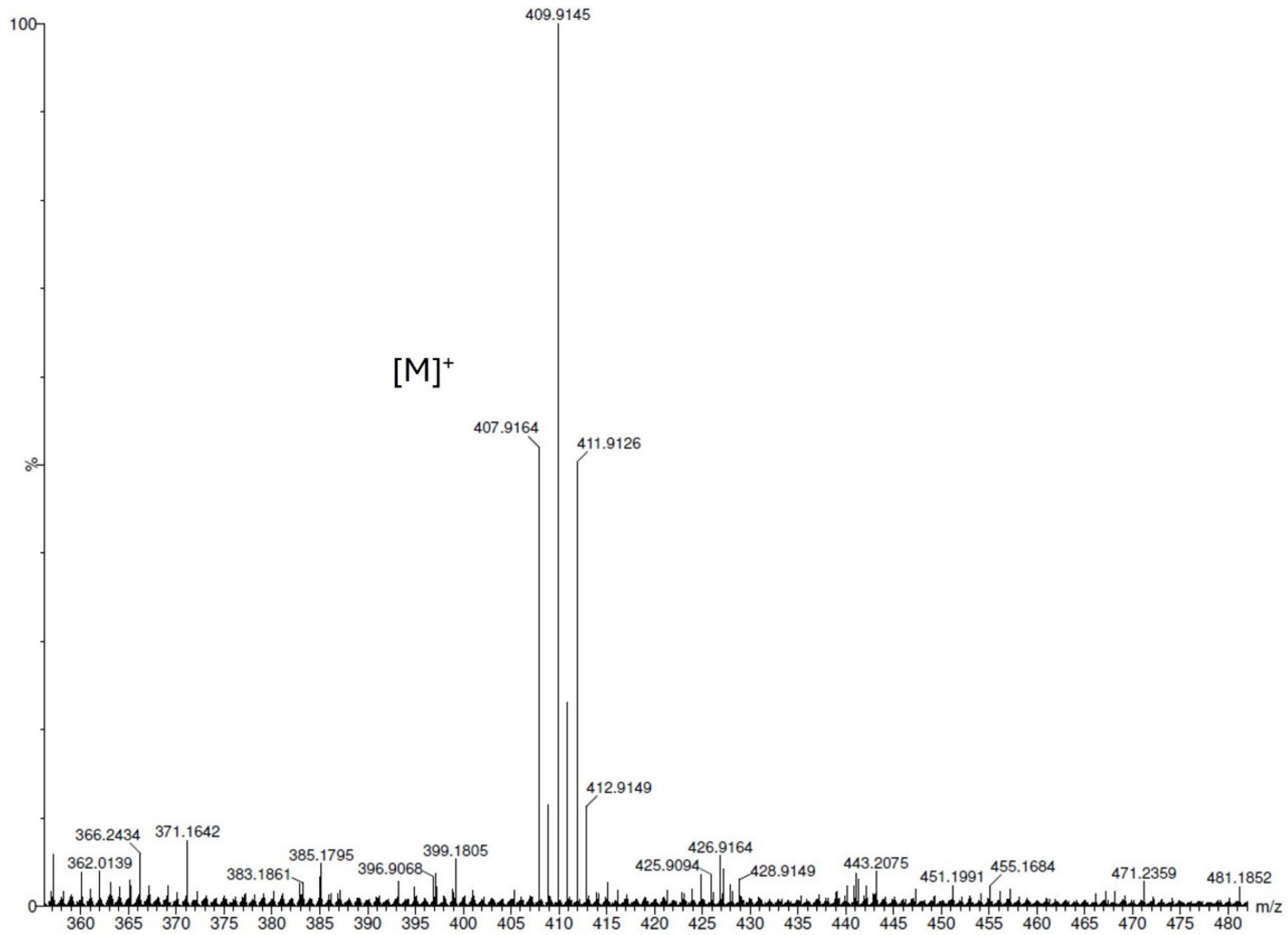

*Figure S17. HRMS (ESI/Q-TOF) spectrum of **755-Prec**.*

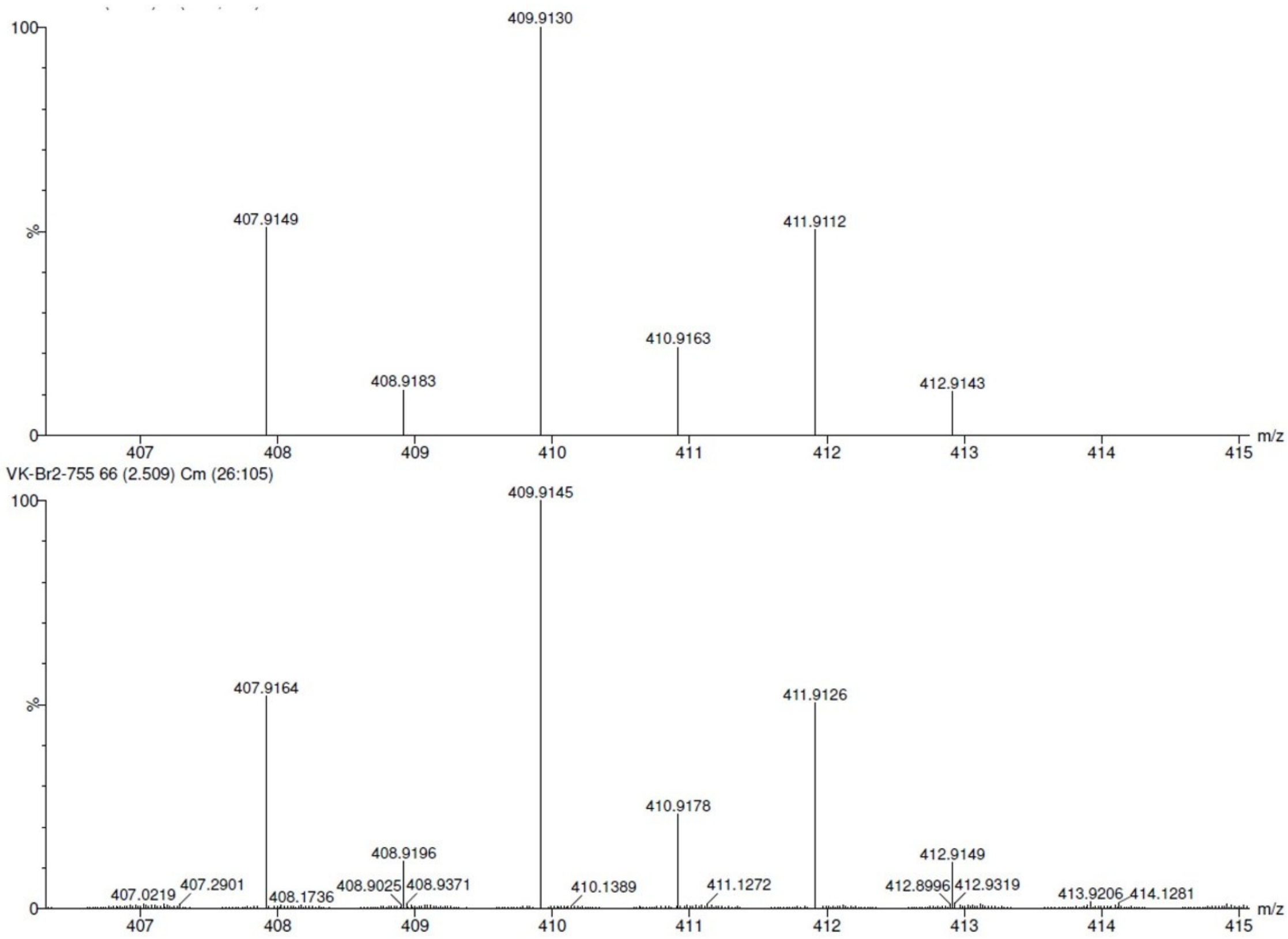

*Figure S18. Observed (bottom) and calculated (top) isotopic distribution for [**755-Prec**]$^+$.*

References

(1) Stengel, M.; Spaldin, N. A.; Vanderbilt, D. Electric Displacement as the Fundamental Variable in Electronic-Structure Calculations. *Nature Phys* **2009**, *5* (4), 304–308. https://doi.org/10.1038/nphys1185.

(2) Toda, T.; Minabe, M.; Yoshida, M.; Tobita, K. Electrophilic Substitution Reactions of Benz[a]Indeno[1,2,3-Cd]Azulene. *J. Org. Chem.* **1990**, *55* (4), 1297–1300. https://doi.org/10.1021/jo00291a037.
